# TRIM24 Cooperates with Ras Mutation to Drive Glioma Progression through snoRNA Recruitment of PHAX and DNA‐PKcs

**DOI:** 10.1002/advs.202400023

**Published:** 2024-06-03

**Authors:** Chenxin Xu, Guoyu Chen, Bo Yu, Bowen Sun, Yingwen Zhang, Mingda Zhang, Yi Yang, Yichuan Xiao, Shi‐Yuan Cheng, Yanxin Li, Haizhong Feng

**Affiliations:** ^1^ State Key Laboratory of Systems Medicine for Cancer Renji‐Med X Clinical Stem Cell Research Center Ren Ji Hospital Shanghai Cancer Institute School of Medicine Shanghai Jiao Tong University Shanghai 200127 China; ^2^ Pediatric Translational Medicine Institute Department of Hematology & Oncology Shanghai Children's Medical Center School of Medicine Shanghai Jiao Tong University National Health Committee Key Laboratory of Pediatric Hematology & Oncology Shanghai 200127 China; ^3^ CAS Key Laboratory of Tissue Microenvironment and Tumor Shanghai Institute of Nutrition and Health University of Chinese Academy of Sciences Chinese Academy of Sciences Shanghai 200031 China; ^4^ Department of Neurology Lou and Jean Malnati Brain Tumor Institute The Robert H. Lurie Comprehensive Cancer Center Simpson Querrey Institute for Epigenetics Northwestern University Feinberg School of Medicine Chicago IL 60611 USA

**Keywords:** DNA‐PKcs, epithelioid GBM, glioma, HRas, PHAX, snoRNA, TRIM24

## Abstract

The factors driving glioma progression remain poorly understood. Here, the epigenetic regulator TRIM24 is identified as a driver of glioma progression, where TRIM24 overexpression promotes HRas^V12^ anaplastic astrocytoma (AA) progression into epithelioid GBM (Ep‐GBM)‐like tumors. Co‐transfection of TRIM24 with HRas^V12^ also induces Ep‐GBM‐like transformation of human neural stem cells (hNSCs) with tumor protein p53 gene (*TP53)* knockdown. Furthermore, TRIM24 is highly expressed in clinical Ep‐GBM specimens. Using single‐cell RNA‐sequencing (scRNA‐Seq), the authors show that TRIM24 overexpression impacts both intratumoral heterogeneity and the tumor microenvironment. Mechanically, HRas^V12^ activates phosphorylated adaptor for RNA export (PHAX) and upregulates U3 small nucleolar RNAs (U3 snoRNAs) to recruit Ku‐dependent DNA‐dependent protein kinase catalytic subunit (DNA‐PKcs). Overexpressed TRIM24 is also recruited by PHAX to U3 snoRNAs, thereby facilitating DNA‐PKcs phosphorylation of TRIM24 at S767/768 residues. Phosphorylated TRIM24 induces epigenome and transcription factor network reprogramming and promotes Ep‐GBM‐like transformation. Targeting DNA‐PKcs with the small molecule inhibitor NU7441 synergizes with temozolomide to reduce Ep‐GBM tumorigenicity and prolong animal survival. These findings provide new insights into the epigenetic regulation of Ep‐GBM‐like transformation and suggest a potential therapeutic strategy for patients with Ep‐GBM.

## Graphical abstract

HRas^V12^ activates PHAX and upregulates U3 snoRNA to recruit Ku‐dependent DNA‐PKcs. Overexpressed TRIM24 is recruited by activated PHAX to U3 snoRNAs, thereby facilitating DNA‐PKcs phosphorylation of TRIM24 protein at S767/768 residues. Phosphorylated TRIM24 induces epigenome and transcription factor network reprogramming and consequently promotes HRas^V12^ anaplastic astrocytoma transforming into Epithelioid Glioblastoma‐like tumors.

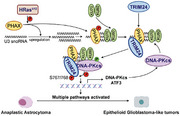

## 1 Introduction

Gliomas are the most common malignant primary intracranial tumors in adults with a poor prognosis and ineffective therapeutic options.^[^ ^1^, ^2^, ^3^ ^]^ Collective analyses of genetic, epigenetic, and proteomic landscapes, as well as single‐cell RNA profiling have identified multiple molecular subgroups with putative prognostic or predictive significance.^[^ ^4^, ^5^ ^]^ However, the poor prognosis is compounded by the endemic problem of glioma progression.

Glioma progression is controlled by the interplay between genetic and non‐genetic factors.^[^ ^6^, ^7^ ^]^ Among the genetic factors, dysregulation of MAPK/PI3K signaling, *EGFR* amplification, and TGF‐β/NF‐κB activation have all been demonstrated to be involved in glioma progression and tumor heterogeneity.^[^ ^8^, ^9^ ^]^ Harvey Rat Sarcoma Viral Oncogene Homolog (HRas) is a member of the RAS proteins family and is highly activated in glioblastoma (GBM).^[^ ^10^, ^11^ ^]^ HRas^V12^ mutation combined with tumor protein p53 gene (TP53) mutation or other gene alterations can induce highly heterogeneous brain tumors;^[^ ^12^, ^13^, ^14^ ^]^ however, the underlying mechanisms remain unclear.

Among epigenetic regulators, histone readers are proteins with structural domains that “read” histone modifications and selectively bind to specific histone post‐translational modifications (PTMs).^[^ ^15^ ^]^ The tripartite motif containing 24 (TRIM24), also known as TIFα, is a member of the tripartite motif (TRIM) family and binds to a specific signature of histone PTMs (H3K4me0/H3K23ac) via a combinatorial Plant Homeo‐domain (PHD) and Bromo‐domain (BRD).^[^ ^16^, ^17^, ^18^ ^]^ Listed as one of the 568 cancer driver genes identified across 66 cancer types,^[^ ^19^ ^]^ TRIM24 has been proven aberrantly expressed and activated in several human cancers, such as breast, prostate, and gastric cancers.^[^ ^20^ ^]^ TRIM24 was also identified to be important for GBM progression, not only by promoting the self‐renewal capacity and invasive growth of glioma stem cells (GSCs) by activating Sox2 expression but also by functioning as a transcriptional co‐activator of STAT3 in *EGFR*‐driven GBM tumorigenesis.^[^ ^21^, ^22^ ^]^ In addition, TRIM24 functions as an E3 ubiquitin ligase targeting p53 for degradation^[^ ^23^ ^]^ and is required for macrophage polarization.^[^ ^24^ ^]^ Conditional TRIM24 overexpression in mouse mammary epithelia induces spontaneous development of metaplastic breast cancer.^[^ ^15^ ^]^ However, the role of TRIM24 in glioma progression and heterogeneity requires further investigation.

DNA‐dependent protein kinase catalytic subunit (DNA‐PKcs) is a pleiotropic serine‐threonine protein kinase that plays a critical role in development and cancer.^[^ ^25^ ^]^ The most extensively investigated role of DNA‐PKcs is as a key regulator of DNA double‐strand break (DSB) repair through non‐homologous end joining (NHEJ).^[^ ^26^ ^]^ In the NHEJ, the Ku70/Ku80 heterodimer binds to the end of the DSB and recruits DNA‐PKcs to form the DNA‐PK holoenzyme.^[^ ^27^ ^]^ In addition to DNA, the Ku heterodimer also drives the assembly of DNA‐PKcs on RNA during ribosome biogenesis.^[^ ^28^ ^]^ Abnormal expression or activation of DNA‐PKcs has been identified as being tightly linked to poor outcomes in multiple hematologic and solid tumors, including gliomas.^[^ ^25^, ^29^, ^30^ ^]^ Pharmacological inhibition of DNA‐PKcs with NU7441 reduced intracranial human GBM xenograft tumor growth and sensitized GBM xenografts to radiotherapy.^[^ ^29^ ^]^ Thus, DNA‐PKcs is a potential targetable pro‐tumorigenic protein kinase in glioma.

Recently, the World Health Organization (WHO) classification of Central Nervous System (CNS) tumors has paid increasing attention to the molecular parameters underlying tumor subtypes and added a new classification of GBM, epithelioid glioblastoma (Ep‐GBM), in 2016.^[^ ^31^ ^]^ Characterized by large epithelioid cells with abundant eosinophilic cytoplasm and melanoma cell‐resembling nucleoli, Ep‐GBM is highly malignant, with a median Overall Survival (OS of 11 months) and progression‐free survival (PFS of 7 months),^[^ ^32^, ^33^, ^34^ ^]^ therefore, exploring the underlying mechanisms of Ep‐GBM formation is a major clinical unmet need. In this study, we report a novel role for TRIM24 in Ep‐GBM‐like tumor formation. By screening a group of TRIM proteins, we found that ectopic expression of TRIM24 cooperated with HRas^V12^ to drive the transformation of human anaplastic astrocytoma (AA) into Ep‐GBM‐like tumors. TRIM24 is also validated to be highly expressed in clinical Ep‐GBM specimens, where co‐transfection with TRIM24 and HRas^V12^ also induced the Ep‐GBM‐like transformation of human neural stem cell (hNSC)‐*TP53* shRNA (hNSC/shTP53) cells. We further describe the mechanism of Ep‐GBM‐like transformation and derive a new therapeutic strategy for the treatment of this clinical subtype of GBM.

## 2 Results

### 2.1 TRIM24 Drives Glioma Progression by Promoting Epithelioid GBM‐Like Transformation

**Figure 1:**
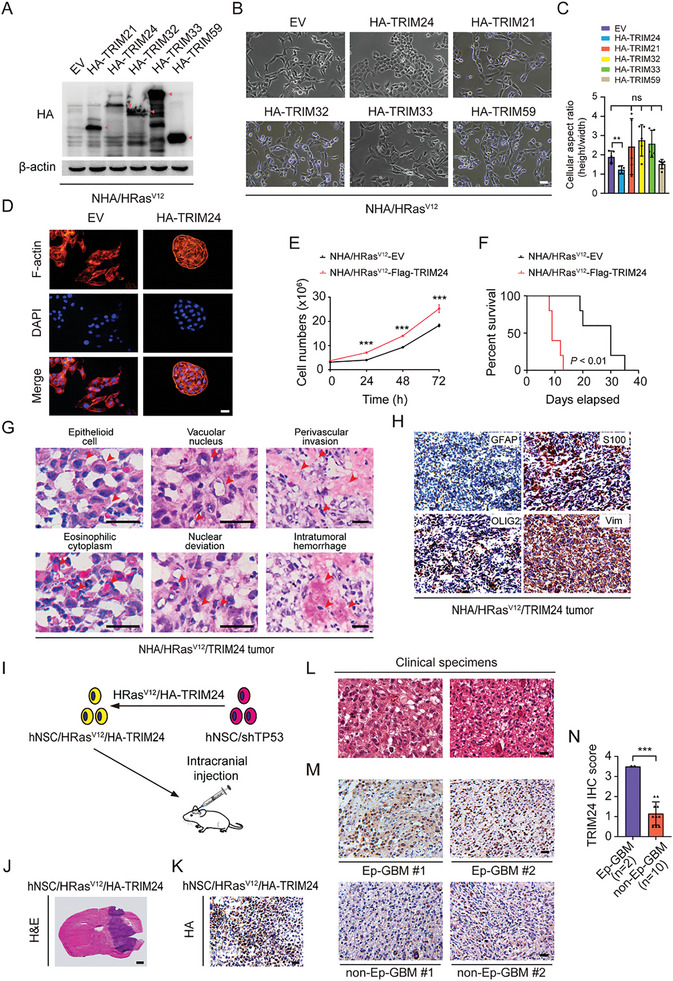
Ectopic expression of TRIM24 drives Ep‐GBM‐like transformation from HRas^V12^ anaplastic astrocytoma. A) Western blot (WB) of ectopic expression of TRIM family proteins in E6/E7/hTERT/HRas^V12^ NHA (NHA/HRas^V12^) cells. EV, empty vector. B) Representative images of morphological changes of NHA/HRas^V12^ cells transfected with indicated TRIM proteins. Scale bar, 100 µm. C) Quantification of differences in the cell aspect ratio of cells in (B). D) Phalloidin staining of NHA/HRas^V12^ cells with or without TRIM24 overexpression. Scale bar, 60 µm. E) Effect of TRIM24 overexpression on NHA/HRas^V12^ cell proliferation. F) Kaplan‐Meier survival analysis of animals bearing NHA/HRas^V12^/EV or NHA/HRas^V12^/TRIM24 brain tumor xenografts (*n* = 5). Median survival (days): EV (30), TRIM24 (9). G) H&E staining of brain tumor xenograft sections. Scale bar, 200 µm. H) IHC analysis of indicated protein expression in xenograft tumors. Scale bar, 25 µm. I) Schematic of the hNSCs transfected with HRas^V12^/HA‐TRIM24 lentiviral‐induced mouse model of Ep‐GBM‐like tumors. Lentivirus‐mediated HRas^V12^ mutant and HA‐TRIM24 were co‐transfected into hNSC‐TP53 shRNA (hNSC/shTP53) cells and then hNSCs were injected into the brains of mice. J) H&E staining of brain tumor xenograft sections. Scale bar, 800 µm. K) IHC analysis of HA‐tag expression in xenograft tumors. Scale bar, 25 µm. L) H&E staining analysis of two clinical Ep‐GBM specimens. Scale bar, 25 µm. M) IHC of TRIM24 in two clinical Ep‐GBM and two representative non‐Ep‐GBM specimens. Scale bar, 25 µm. N) Quantification of TRIM24 expression in panel (M). Data represent two or three independent experiments with similar results. Error bars, s.d. ns indicates not significant. *P < 0.05, **P < 0.01, ***P < 0.001, by two‐tailed Student's t‐test, one‐way ANOVA analysis or log‐rank analysis.

To screen for putative TRIM proteins that function as epigenetic regulators of glioma progression, we overexpressed a group of TRIM family proteins, including TRIM24, in HRas^V12^‐expressing immortalized normal human astrocyte (NHA)‐E6/E7/hTERT cells (E6/E7, to inactivate both p53 and pRb), which were previously shown to form intracranial tumors in mice resembling WHO grade 3 AA.^[^ ^35^ ^]^ In addition, NHAs appear to be more genetically stable than rodent astrocytes.^[^ ^35^, ^36^ ^]^ After drug selection, only the morphology of TRIM24‐overexpressing NHA/HRas^V12^ cells changed morphologically into more epithelial‐like cells (**Figure** **1**A–C), which were characterized by a cuboidal shape and a dense arrangement. Cells overexpressing TRIM21, TRIM32, TRIM33, or TRIM59 remained unchanged (Figure 1A–C). Phalloidin staining of the cytoskeleton further showed that the cell protrusions had vanished, whereas the cell bodies changed into flat and irregular polygonal shapes. After division, the NHA/HRas^V12^/TRIM24 cells tended to cluster together rather than disperse (Figure 1D), and the ectopic expression of TRIM24 promoted cell proliferation (Figure 1E). To investigate whether TRIM24 overexpression specifically induced NHA/HRas^V12^ cells transformation, we overexpressed TRIM24 in NHAs and found no obvious changes in cell morphology (Figure S1A–C, Supporting Information).

To further investigate whether ectopic TRIM24 expression in NHA/HRas^V12^ cells could drive Ep‐GBM‐like transformation from AA in vivo, we stereotactically implanted NHA/HRas^V12^/TRIM24 or NHA/HRas^V12^/EV control cells into mouse brains. Consistent with the increased cell growth in vitro, animals with NHA/HRas^V12^/TRIM24 brain tumor xenografts showed significantly shortened survival (Figure 1F). Hematoxylin‐eosin (H&E) staining analysis revealed that, compared to NHA/HRas^V12^/EV tumors, the tumor cytology in NHA/HRas^V12^/TRIM24‐derived tumors presented as a high‐grade glioma with heteromorphic large epithelioid cells with abundant eosinophilic cytoplasm, a high degree of nuclear atypia, and laterally positioned melanoma‐like nuclei. The borders of the tumors were discrete and full of infiltrating angiogenic cells (Figure 1G). NHA/HRas^V12^/TRIM24‐derived tumors strongly expressed S100 and Vimentin proteins, whereas the expression of GFAP and OLIG2 proteins was limited (Figure 1H). These data suggest that NHA/HRas^V12^/TRIM24‐derived tumors resemble Ep‐GBM in the clinical diagnostic criteria of Ep‐GBM summarized in the 2016 WHO Classification of CNS Tumors and previously reported descriptions of Ep‐GBM.^[^ ^31^, ^37^, ^38^, ^39^ ^]^ To further validate the ability of TRIM24 to induce Ep‐GBM‐like formation in vivo, we co‐transfected lentivirus‐mediated HRas^V12^ mutant and HA‐TRIM24 into hNSC‐*TP53* shRNA (hNSC/shTP53) cells (Figure 1I). Mice that injected with these modified hNSCs cells intracranially also developed brain tumors with the aforementioned pathophysiological features resembling those of Ep‐GBM (Figure 1J,K).

We further compared the TRIM24 expression levels in two clinical Ep‐GBM and ten clinical non‐Ep‐GBM specimens using immunohistochemistry (IHC) analysis. As shown in Figure 1L–N, Ep‐GBM tumors showed remarkably higher TRIM24 expression than non‐Ep‐GBM tumors. Taken together, our data suggests that the epigenetic regulator TRIM24 can function as a driver of Ep‐GBM‐like tumors transformation and a regulator of glioma progression.

### 2.2 scRNA‐Sequencing Indicates that TRIM24 Overexpression Markedly Impacts both Intratumoral Heterogeneity and the Tumor Microenvironment

**Figure 2:**
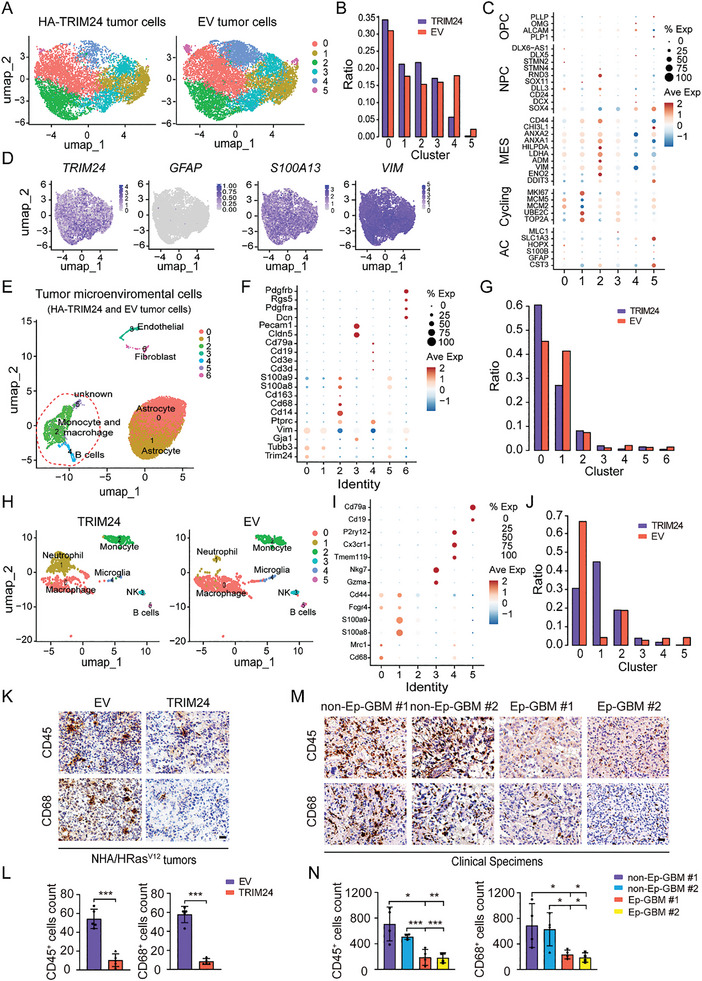
scRNA‐sequencing indicates that TRIM24 overexpression markedly impacts both intratumoral heterogeneity and the tumor microenvironment. A) Uniform manifold approximation and projection (UMAP) visualization of all 9280 cells from TRIM24‐triggered brain tumors and all 10892 cells from EV control brain tumors. B) Composition ratio of the six clusters in TRIM24‐triggered and EV‐triggered brain tumors. C) Dot plot showing marker gene expression for different glioma subtypes. The dot sizes indicate the percentage of cells in each cluster expressing the gene and the colors indicate average expression levels. D) UMAP projection of TRIM24‐triggered brain tumor cells displaying representative marker gene expression. The intensity of color indicated the average expression levels. E) UMAP visualizations of cells infiltrated in TRIM24‐triggered brain tumors. F) Dot plots showing marker gene expression for different cell types. Dot size indicate the percentage of cells in each cluster expressing the gene and the colors indicate average expression levels. G) Composition ratio of the seven clusters in TRIM24‐triggered and EV‐triggered brain tumors. H) UMAP visualizations of immune cells infiltrated in TRIM24‐triggered and EV‐triggered brain tumors. I) Dot plots showing marker gene expression for different immune cell types. J) Composition ratio of the six clusters in TRIM24‐triggered and EV‐triggered brain tumors. K) Representative images of CD45 and CD68 IHC staining in brain cross sections from NHA/HRas^V12^/EV‐derived and NHA/HRas^V12^/TRIM24‐derived mouse tumors. Scale bar: 25 µm. L) Quantification of CD45 and CD68 positive cells in (K). M) Representative images of CD45 and CD68 IHC staining in brain cross sections of clinical GBM and Ep‐GBM patient specimens. Scale bar: 25 µm. N) Quantitation of CD45 and CD68 positive cells in (M). Data represent two or three independent experiments with similar results. Error bars, s.d. *P < 0.05, **P < 0.01, ***P < 0.001, by two‐tailed Student's *t*‐test.

To investigate the cellular heterogeneity and molecular features of NHA/HRas^V12^/TRIM24‐derived Ep‐GBM‐like tumors, we performed single‐cell RNA sequencing (scRNA‐Seq) analysis of NHA/HRas^V12^/TRIM24 and NHA/HRas^V12^ control xenograft tumors formed in mice brains. Unsupervised cluster analysis based on RNA expression revealed six clusters with distinct gene expression patterns. Compared to the control, the proportions of clusters 4 and 5 decreased in HRas^V12^/TRIM24 tumors while the other three clusters were quite similar (**Figure** **2**A,B). We then performed single‐sample gene set enrichment analysis (ssGSEA) with gene meta‐modules^[^ ^5^ ^]^ to identify the cellular states of the six clusters, including oligodendrocyte progenitor‐like (OPC‐like), neural progenitor‐like (NPC‐like), mesenchymal‐like (MES‐like), astrocyte‐like (AC‐like), and proliferating population (cycling). Compared to the control tumors, which were primarily composed of OPC‐like and AC‐like cells, HRas^V12^/TRIM24 tumors were enriched with MES‐like and, NPC‐like cells, as well as, a proliferating population (Cycling) (Figure 2C). Consistent with the Ep‐GBM‐specific markers shown in Figure 1H, *TRIM24*, *S100*, and *Vim* were highly expressed in HRas^V12^/TRIM24 tumor cells, whereas *GFAP* was expressed at markedly lower levels (Figure 2D). These data indicated that TRIM24 overexpression affects intratumoral heterogeneity.

Since tumor‐intrinsic transcriptional subtypes correlate with tumor microenvironment (TME) heterogeneity,^[^ ^40^ ^]^ we further compared TME heterogeneity between HRas^V12^/TRIM24 and control tumors. As shown in Figure 2E,F, specific gene sets were used to distinguish the clusters, and seven clusters were identified, including two distinct astrocyte clusters, one cluster of monocytes and macrophages, one cluster of endothelial cells, one cluster of B cells, and one cluster of fibroblast cells. The proportions of clusters 0 and 1 (astrocytes) in the two groups were higher than those of the other clusters in both tumors (Figure 2G). Further identification of these TME cells (clusters 2, 4, and 5 in Figure 2E) indicated that compared to the control, the ratio of macrophages was reduced in HRas^V12^/TRIM24 tumors, whereas the ratio of neutrophils was increased (Figure 2H–J). The lower macrophage infiltration in Ep‐GBM‐like tumor xenografts compared to that in the control was further confirmed by IHC analysis (Figure 2K,L). Consistently, macrophages showed less infiltration in clinical Ep‐GBM specimens than in non‐Ep‐GBM specimens (Figure 2M,N). We compared the polarization of infiltrating macrophages and neutrophils between HRas^V12^/TRIM24 tumors and controls. As shown in Figure S2A,B (Supporting Information), the infiltrated macrophages were identified as four clusters, and the expression levels of the M1 macrophage markers *CD68* and *Irf5* were lower in HRas^V12^/TRIM24 tumors than in the controls (Figure S2C, Supporting Information). The infiltrated neutrophils were identified as two clusters (Figure S2D,E, Supporting Information), and the expression level of the N2 neutrophil marker *Arg2* was higher in HRas^V12^/TRIM24 tumors than the controls (Figure S2F, Supporting Information). Since macrophages and neutrophils are the most enriched innate immune cells in GBM and the M2 and N2 polarized phenotypes, respectively, are associated with immunosuppression,^[^ ^41^, ^42^ ^]^ the altered infiltration of macrophages and neutrophils in Ep‐GBM‐like tumor xenografts may contribute to the high malignancy of this subtype and immunotherapy resistance. Taken together, our scRNA‐sequencing results showed that TRIM24 overexpression markedly affected both intratumoral heterogeneity and the tumor microenvironment.

### 2.3 TRIM24 Drives Ep‐GBM‐Like Transformation through Epigenome and Transcription Factor Network Remodeling

**Figure 3:**
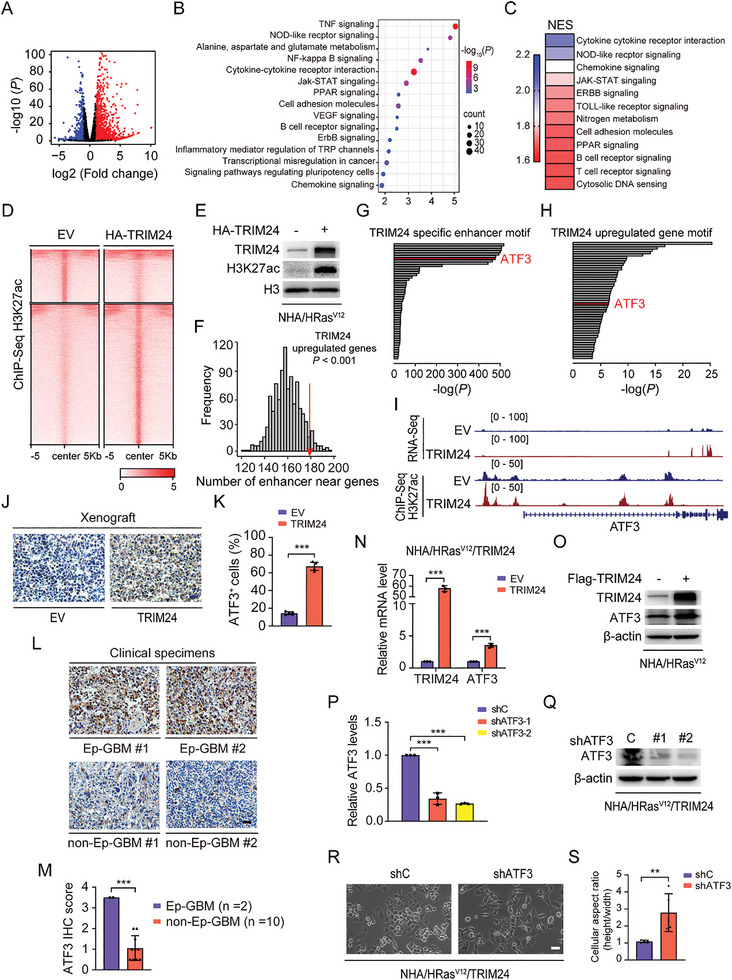
TRIM24 drives Ep‐GBM‐like transformation through epigenome and transcription factor network remodeling. A) Volcano plot depicting up‐ and down‐regulated genes more than 2‐fold after TRIM24 overexpression in NHA/HRas^V12^ cells. B) KEGG pathway analysis of the DEGs in NHA/HRas^V12^/TRIM24 cells. C) GSEA analysis of RNA Sequencing data revealed 12 gene sets significantly enriched in NHA/HRas^V12^/TRIM24 cells (*FDR* < 0.25, *P* < 0.01). The gene sets are colored according to the normalized enrichment score (*NES*). D) Heatmap of H3K27ac ChIP‐Seq showing effects of TRIM24 overexpression on H3K27ac enrichment compared to the empty vector (EV) in NHA/HRas^V12^ cells. E) WB of the effect of TRIM24 overexpression on H3K27 acetylation in NHA/HRas^V12^ cells. F) TRIM24‐upregulated genes are located near TRIM24‐responsive enhancers. G) Motif enrichment analysis indicated that *ATF3* is enriched at TRIM24‐specific enhancers. H) Motif enrichment analysis indicates that *ATF3* is enriched at TRIM24‐upregulated genes. I) RNA‐Seq and ChIP‐Seq overlay tracks of H3K27ac marks for the gene *ATF3* in NHA/HRas^V12^/EV and NHA/HRas^V12^/TRIM24 cells. J) IHC analysis of ATF3 protein expression in NHA/HRas^V12^/EVand NHA/HRas^V12^/TRIM24^WT^ xenograft tumors. Scale bar, 25 µm. K) Quantification of ATF3 positive cells in (J). L) IHC analysis of ATF3 expression in two clinical Ep‐GBM and two representative non‐Ep‐GBM specimens. Scale bar, 25 µm. M) Quantification of ATF3 expression in panel (L). N) QRT‐PCR of effects of TRIM24 overexpression on *ATF3* mRNA expression. O) WB of effects of TRIM24 overexpression on ATF3 expression in NHA/HRas^V12^ cells. P) QRT‐PCR of *ATF3* knockdown (KD) in NHA/HRas^V12^/TRIM24 cells. Q) WB of *ATF3* KD with two different shRNAs (shATF3‐1 and shATF3‐2) or a control shRNA in NHA/HRas^V12^/TRIM24 cells. R) Representative images showing the effects of *ATF3* KD on NHA/HRas^V12^/TRIM24 cell morphology. Scale bar, 100 µm. S) Quantification of the differences in the cell aspect ratio of cells in (R). Data represent two or three independent experiments with similar results. Error bars, s.d. **P < 0.01, ***P < 0.001, by two‐tailed Student's *t*‐test.

To understand the mechanism by which TRIM24 drives AA transformation toward Ep‐GBM, we performed bulk RNA‐seq analysis of NHA/HRas^V12^ cells transfected with or without TRIM24. A total of 1421 differentially expressed genes (DEGs) were identified according to the cut‐off criteria (fold change > 2, *P* < 0.05) in NHA/HRas^V12^/TRIM24 cells, among which 1104 genes were upregulated and 317 genes were downregulated (**Figure** **3**A). To elucidate the possible pathways TRIM24‐activated that may be involved in AA transformation toward Ep‐GBM, we performed KEGG pathway analysis and GSEA analysis of these 1104 upregulated genes and found that multiple pathways were highly activated in the NHA/HRas^V12^/TRIM24 cells, such as the TNF, MAPK, and PPAR signaling pathway (Figure 3B,C; Figure S3A, Supporting Information). Given that TRIM24 functions as a transcriptional co‐activator in breast cancer, prostate cancer, and GBM,^[^ ^18^, ^20^, ^22^ ^]^ we reasoned that TRIM24 activated these pathways by regulating the transcriptional activation of multiple regulators in these pathways.

We then determined how TRIM24 simultaneously induces the transcriptional activation of these pathway regulators. Recently, the development of epigenomics has revealed that epigenetic markers on chromatin are important signatures of enhancers that cooperate with transcription factors and are involved in the transcriptional regulation of many genes in various types of cancers.^[^ ^43^, ^44^ ^]^ Since histone modifications are widely used for the identification of enhancers on chromatin,^[^ ^44^, ^45^ ^]^ we examined multiple H3 modification markers. As shown in Figure S3B (Supporting Information), TRIM24 overexpression significantly increased H3K27 acetylation (H3K27ac), whereas the expression of other histone modifications showed a minimal increase or were unchanged. To investigate whether there was an increase in enhancer activity in NHA/HRas^V12^/TRIM24 cells, we performed H3K27ac Chromatin Immunoprecipitation Sequencing (ChIP‐Seq) in NHA/HRas^V12^ cells transfected with TRIM24 or an EV control. As shown in Figure 3D,E, we identified active enhancers in NHA/HRas^V12^/TRIM24 cells by increased H3K27ac peaks far from the transcription start sites (TSS).

To identify which genes achieved a high H3K27ac signal in their enhancers to increase transcription, we combined our RNA‐Seq and ChIP‐Seq analyses and found that enhancers were enriched around TRIM24‐specific upregulated genes (Figure 3F). Among these upregulated genes, activating transcription factor 3 (ATF3) was noted because enrichment of TRIM24 specific enhancer motif was shown in *ATF3*, accompanied by elevation of the H3K27ac signal on its enhancer (Figure 3G–I). Given that ATF3 functions in multiple pathways to regulate cancer progression,^[^ ^46^, ^47^ ^]^ we proposed that ATF3 is a key downstream gene regulated by TRIM24 in the TRIM24‐driven Ep‐GBM‐like transformation.

To test this hypothesis, we first analyzed ATF3 protein expression in HRas^V12^/TRIM24 brain tumor xenografts and clinical Ep‐GBM samples. As shown in Figure 3J,K, higher ATF3 expression was detected in NHA/HRas^V12^/TRIM24‐derived tumors than NHA/HRas^V12^/EV‐derived tumors. Analysis of ATF3 expression in clinical Ep‐GBM tumors also showed a remarkably higher signal than in non‐Ep‐GBM tumors (Figure 3L,M). Consistent with the RNA‐Seq data, in vitro experiments showed increased *ATF3* mRNA expression in NHA/HRas^V12^/TRIM24 cells (Figure 3N). ATF3 protein levels were also consistently increased in NHA/HRas^V12^/TRIM24 cells as compared to those in the control (Figure 3O). To further investigate the link between *ATF3* and *TRIM24* expression, we validated their correlations using the CGGA and Rembrandt databases. As shown in Figure S3C (Supporting Information), a clear positive correlation was observed between *ATF3* and *TRIM24* expression levels. All these data suggest that TRIM24 serves as a transcriptional regulator of ATF3 rather than as an E3 ubiquitin ligase. After *ATF3* knock down (KD) in NHA/HRas^V12^/TRIM24 cells, the TRIM24‐driven Ep‐GBM‐like transformation was markedly diminished in these *ATF3*‐KD cells (Figure 3P–S), indicating that ATF3 plays a significant role in the TRIM24‐dependent transformation. Collectively, these data suggest that elevated TRIM24 expression in NHA/HRas^V12^ cells induces epigenome and transcription factor network remodeling to drive Ep‐GBM‐like transformation.

### 2.4 DNA‐PKcs is Required for TRIM24‐Driven Ep‐GBM‐Like Transformation

**Figure 4:**
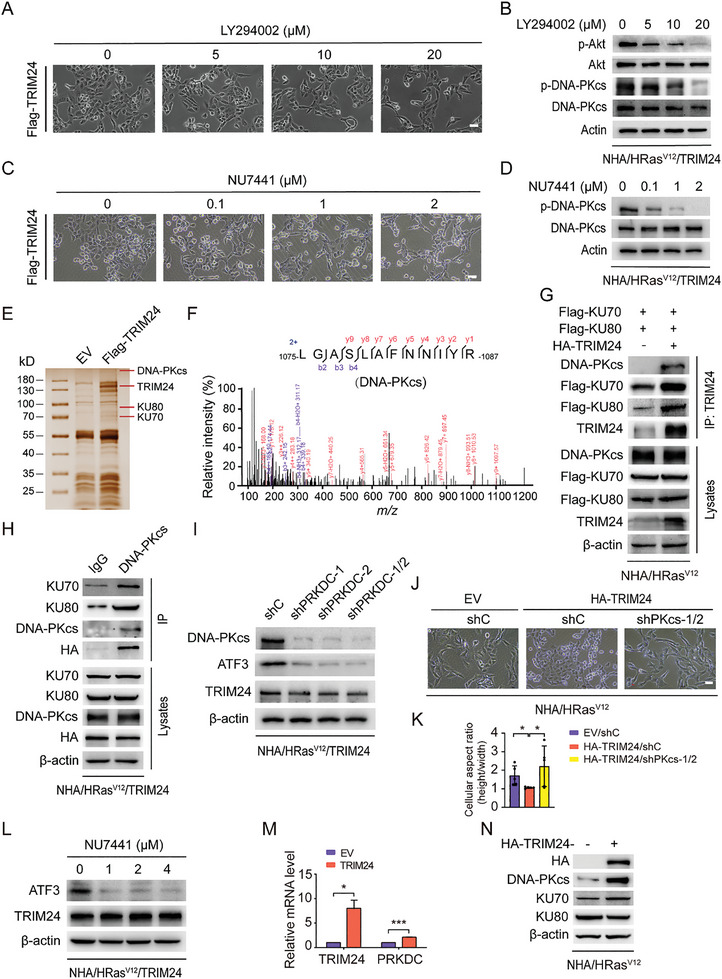
Ku‐dependent DNA‐PKcs is required for TRIM24‐driven Ep‐GBM‐like transformation. A) Morphological changes of NHA/HRas^V12^/TRIM24 cells treated with LY294002 for 24 h at indicated concentrations. Scale bar, 100 µm. B) WB of the inhibitory effects of LY294002 on AKT phosphorylation and DNA‐PKcs phosphorylation in cells in (A). C) Morphological changes of NHA/HRas^V12^/TRIM24 cells treated with NU7441 for 24 h at indicated concentrations. Scale bar, 100 µm. D) WB of the inhibitory effects of NU7441 on DNA‐PKcs phosphorylation in cells in (C). E) Silver staining of an SDS PAGE gel separating TRIM24‐associated proteins in NHA/HRas^V12^/TRIM24 cells. F) DNA‐PKcs peptides in the TRIM24‐interacting proteins immunoprecipitated from NHA/HRas^V12^/TRIM24 cells and identified by mass spectrometry analysis. G) Immunoprecipitation (IP) and WB analysis of TRIM24 association with the DNA‐PK complex (Ku70, Ku80, and DNA‐PKcs) in NHA/HRas^V12^/TRIM24 cells. H) Co‐IP analysis of protein interactions between TRIM24 and the DNA‐PK complex. I) WB of the effects of *PRKDC* KD on ATF3 expression with two different shRNAs (shPKcs‐1 and shPKcs‐2) or double shRNAs (shPKcs‐1/2). shC, a control shRNA. J) Representative images showing the effects of *PRKDC* KD on NHA/HRas^V12^/TRIM24 cell morphology. Scale bar, 100 µm. K) Quantification of the differences in the cell aspect ratio of cells in (J). L) WB of the inhibitory effects of NU7441 on ATF3 expression. Cells were treated with NU7441 at indicated concentrations for 24 h. M) QRT‐PCR of effects of TRIM24 overexpression on *PRKDC* mRNA expression. N) WB of the DNA‐PK complex expression with TRIM24 overexpression in NHA/HRas^V12^ cells. Data represent two or three independent experiments with similar results. Error bars, s.d. *P < 0.05, ***P < 0.001, by two‐tailed Student's *t*‐test.

Since RAS‐RAF‐MEK‐ERK and RAS‐PI3K‐AKT‐ mTOR act as fundamental signaling pathways for RAS proteins,^[^ ^48^, ^49^, ^50^ ^]^ we determined whether the PI3K inhibitor LY294002, the MEK inhibitor U0126, or the mTOR inhibitor rapamycin could impair TRIM24‐induced Ep‐GBM‐like transformation. As shown in **Figure** **4**A,B, and Figure S4A,C–F (Supporting Information), treatment with the PI3K inhibitor LY294002 markedly impaired the TRIM24‐driven Ep‐GBM‐like transformation compared to the control. To explore whether PI3K is the key kinase, we further treated NHA/HRas^V12^/TRIM24 cells with or without the PI3K p110α inhibitor PIK‐75, PI3K p110β inhibitor TGX‐221, and the AKT inhibitor MK2206, respectively. However, none of these inhibitors reduced the transformation induced by TRIM24, indicating that PI3K is not a functional kinase in this process (Figure S4G–L, Supporting Information) and that another unknown molecule inhibited by LY294002 is involved in Ep‐GBM‐like transformation. Since LY294002 is also a competitive inhibitor of DNA‐PKcs^[^ ^51^ ^]^ we treated NHA/HRas^V12^/TRIM24 cells with a highly specific DNA‐PKcs inhibitor, NU7441, and found that TRIM24‐induced Ep‐GBM‐like transformation was markedly diminished (Figure 4C,D; Figure S4B, Supporting Information). These data suggested that DNA‐PKcs may play a key role in the TRIM24‐driven Ep‐GBM‐like transformation.

To test our hypothesis, we purified the TRIM24 complex using an anti‐FLAG antibody pull‐down from NHA/HRas^V12^/TRIM24 or control cells followed by mass spectrometry (Figure 4E). We found that TRIM24 interacts with components of the DNA‐PK complex, including DNA‐PKcs, Ku70, and Ku80, in NHA/HRas^V12^ cells (Figure 4F–H; Figure S5A,B, Supporting Information). To determine whether DNA‐PKcs regulated TRIM24‐inducing Ep‐GBM‐like transformation, we knocked down *PRKDC* in NHA/HRas^V12^/TRIM24 cells (Figure 4I). As expected, the TRIM24‐driven Ep‐GBM‐like transformation was markedly impaired by *PRKDC* KD (Figure 4J,K).

As the data in Figure 3 suggest that ATF3 is a key downstream effector in the process of TRIM24‐driven Ep‐GBM‐like transformation, the effects of DNA‐PKcs inhibition on ATF3 expression in NHA/HRas^V12^/TRIM24 cells were assessed. As shown in Figure 4I, *PRKDC* KD reduced ATF3 protein expression. Consistently, a dose‐dependent inhibition by NU7441 on levels of ATF3 protein and mRNA expression (Figure 4L; Figure S5C, Supporting Information) was also observed. These data indicate that DNA‐PKcs is involved in TRIM24‐driven Ep‐GBM‐like transformation and that its kinase activity is required for TRIM24 regulation of *ATF3* transcription.

Given that DNA‐PKcs directly interact with TRIM24 and is essential for TRIM24‐driven Ep‐GBM‐like transformations, we determined whether there is an expressional regulatory relationship between TRIM24 and DNA‐PKcs. Compared to NHA/HRas^V12^ cells, ectopic expression of TRIM24 markedly increased the levels of DNA‐PKcs protein and mRNA, suggesting that TRIM24 serves as a transcriptional regulator of DNA‐PKcs rather than an E3 ubiquitin ligase (Figure 4M,N). Taken together, these data suggest that DNA‐PKcs is transcriptionally regulated by TRIM24 and that TRIM24‐driven Ep‐GBM‐like transformation is dependent on DNA‐PKcs.

Since DNA‐PKcs is a classical NHEJ factor, we investigated whether TRIM24 overexpression induced DNA damage to activate DNA‐PKcs in our model. As shown in Figure S5D,E (Supporting Information), no obvious increase in human phosphorylated histone (γH2AX) protein levels and γH2AX foci formation was observed along with TRIM24 overexpression, suggesting that the activation of DNA‐PKcs was not triggered by DNA damage in our model and there existed another pathway to induce DNA‐PKcs activation.

### 2.5 DNA‐PKcs Phosphorylates TRIM24 at S767/768 to Promote Ep‐GBM‐Like Transformation

**Figure 5:**
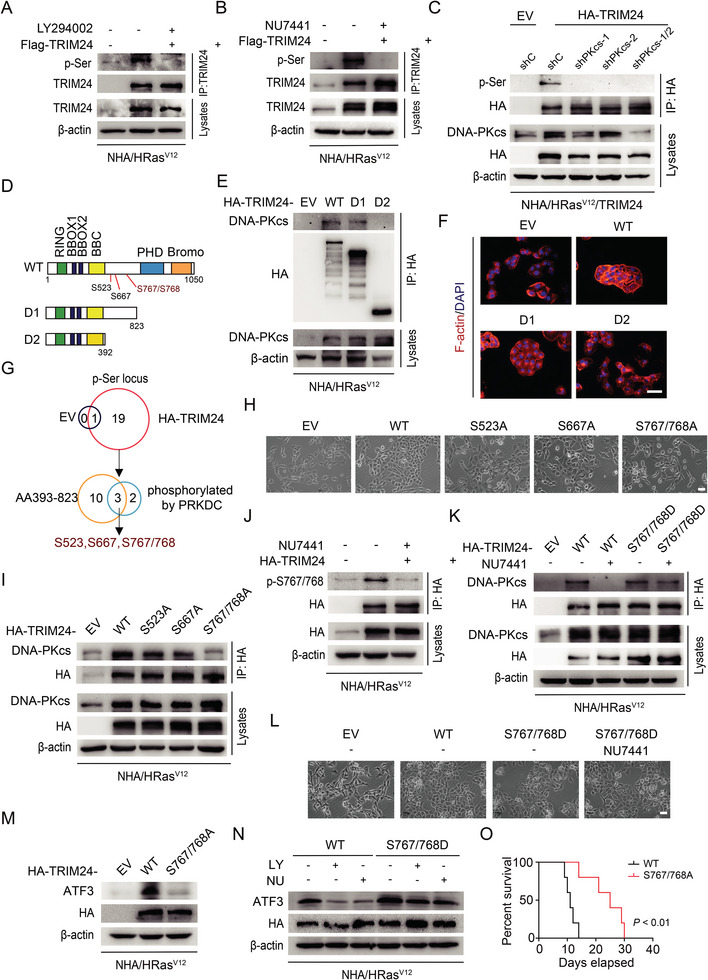
DNA‐PKcs phosphorylates TRIM24 at S767/768 to promote Ep‐GBM‐like transformation. A,B) Inhibitory effects of LY294002 (A) and NU7441 (B) on TRIM24 phosphorylation in NHA/HRas^V12^/TRIM24 cells. Cells were treated with LY294002 (20 µm) or NU7441 (4 µm) for 24 h. C) Effects of *PRKDC* KD on TRIM24 phosphorylation. D) Schematics of TRIM24 wild‐type (WT) and various TRIM24 deletion constructs. E) DNA‐PKcs interacts with TRIM24 at amino acid residues 393–823. TRIM24 WT or the indicated mutants were transfected into NHA/HRas^V12^ cells. F) Phalloidin staining analysis of morphological changes of NHA/HRas^V12^ cells transfected with TRIM24 WT or the indicated mutants. Scale bar, 60 µm. G) Venn diagram depicting distinct phosphorylation sites in TRIM24 (up) and overlapped phosphorylation sites located among amino acid (AA) residues 393–823 between mass spectrometry analysis and silico analysis of sites predicted to be phosphorylated by DNA‐PKcs (down). The location of three overlapped phosphorylated residues S523, S667, and S767/768 are shown in D. H) Representative images of morphological changes of NHA/HRas^V12^ cells transfected with TRIM24 WT, S523A, S667A, and S767/768A mutants, or EV. Scale bar, 100 µm. I) Effects of the TRIM24 mutations S523A, S667A, and S767/768A on TRIM24 binding with DNA‐PKcs in NHA/HRas^V12^ cells. J) Effects of NU7441 on TRIM24 p‐S767/768 in NHA/HRas^V12^/TRIM24 cells. Cells were treated with NU7441 (4 µm) for 24 h. K) Effect of TRIM24 S767/768D mutation on TRIM24 binding with DNA‐PKcs. NHA/HRas^V12^ cells were treated with NU7441 (4 µm) for 24 h. L) Representative images of the effect of the TRIM24 S767/768D mutation on morphological changes of NHA/HRas^V12^ cells treated with NU7441 (4 µm) for 24 h. Scale bar, 100 µm. M) WB of effect of TRIM24 S767/768A mutation on ATF3 expression in NHA/HRas^V12^cells. N) WB analysis of effect of the TRIM24 S767/768D mutation on ATF3 expression compared to TRIM24 WT in NHA/HRas^V12^ cells treated with LY294002 (20 µm) or NU7441 (4 µm) for 24 h. O) Kaplan‐Meier survival analysis of animals bearing NHA/HRas^V12^/TRIM24^WT^ or NHA/HRas^V12^/TRIM24^S767/768A^ tumor xenografts (*n* = 5). Median survival (days): TRIM24^WT^ (11), TRIM24^S767/768A^ (25). Data represent two or three independent experiments with similar results. *P* value was determined using log‐rank analysis.

DNA‐PKcs is a protein kinase involved in various cellular functions through the phosphorylation of substrates, such as AMPK.^[^ ^52^ ^]^ Thus, we hypothesized that DNA‐PKcs could phosphorylate TRIM24 during the Ep‐GBM‐like transformation. We first treated NHA/HRas^V12^/TRIM24 cells with DNA‐PKcs inhibitors and found that phosphorylation of TRIM24 at serine residues (p‐Ser) was markedly reduced in cells treated with LY294002 or NU7441 (**Figure** **5**A,B). Consistently, *PRKDC* KD diminished the p‐Ser level in TRIM24 (Figure 5C). These results supported the hypothesis that DNA‐PKcs regulates TRIM24 serine phosphorylation.

To further identify which region or domain in the TRIM24 protein is associated with DNA‐PKcs, we generated two deletion mutants that lacked the C‐terminal region of amino acid (AA) residues 824–1050 (D1) or 393–1050 (D2) of the TRIM24 protein, respectively (Figure 5D). The mutant D1, but not the mutant D2, was able to bind to DNA‐PKcs (Figure 5E), suggesting that the AA residues 393–823 of TRIM24 are required for its interaction with DNA‐PKcs. Furthermore, NHA/HRas^V12^ cells transfected with mutant D1 showed a similar Ep‐GBM‐like transformation as the TRIM24 wild‐type (WT), but remained unchanged after transfection with mutant D2 (Figure 5F), suggesting that the fragment of AA residues 393–823 of TRIM24 is required for TRIM24‐driven Ep‐GBM‐like transformation.

To assess which serine (S) residues are phosphorylated by DNA‐PKcs and are necessary for Ep‐GBM‐like transformation, we performed mass spectrometric analysis of the TRIM24 protein purified from NHA/HRas^V12^/TRIM24 cells. Among the 19 potentially phosphorylated serine residues, 13 were located in the AA fragment 393–823. We then performed in silico analysis of the consensus AA residues of TRIM24 as a potential serine kinase substrate using GPS 6.0 software (http://gps.biocuckoo.org). Three putative phosphorylation sites, S523, S667, and S767/768 were located in the AA fragment 393–823 of TRIM24 and were predicted to be phosphorylated by DNA‐PKcs (Figure 5G). Non‐phosphorylatable S523A, S667A, and S767/768A mutants were generated and separately transfected into NHA/HRas^V12^ cells. Compared to the TRIM24 WT, S523A, and S667A mutations, only the S767/768A mutation significantly reduced Ep‐GBM‐like transformation (Figure 5H; Figure S6A, Supporting Information). Additionally, the S767/768A mutations, but not the S523A or S667A mutations, markedly reduced the interaction between TRIM24 and DNA‐PKcs (Figure 5I).

IB analysis of NHA/HRas^V12^ cell lysates using a specific phosphorylation antibody for p‐S767/768 of TRIM24 revealed that p‐S767/768 was reduced by NU7441 treatment (Figure 5J). Mass spectrometry analysis confirmed that S767/768 of TRIM24 was phosphorylated (Figure S6B, Supporting Information). Protein sequence alignment analysis showed that the domain containing S767/768 in TRIM24 was conserved among humans and mammalian species (Figure S6C, Supporting Information). We introduced the phosphomimetic mutant S767/768D into NHA/HRas^V12^ cells. As shown in Figure 5K, treatment with NU7441 significantly decreased the interaction between DNA‐PKcs and TRIM24 WT, but not the S767/768D mutant. The S767/768D mutation significantly reversed Ep‐GBM‐like transformation impaired by NU7441 (Figure 5L; Figure S6D, Supporting Information). These data support the idea that the S767/768 residues of TRIM24 are phosphorylated by DNA‐PKcs, and that p‐S767/768 is necessary for TRIM24‐driven Ep‐GBM‐like transformation.

Finally, we assessed the roles of p‐S767/768 in regulating ATF3 expression in vitro and tumor progression in vivo. As shown in Figure 5M, the S767/768A mutation significantly reduced ATF3 protein expression in NHA/HRas^V12^ cells. Additionally, the phosphomimetic S767/768D mutation markedly attenuated the LY294002 or NU7441 inhibition of ATF3 protein expression in these cells (Figure 5N). Compared with animals bearing TRIM24 WT tumor xenografts, animals with NHA/HRas^V12^/TRIM24 S767/768A tumor xenografts showed prolonged survival (Figure 5O). Medium expression of GFAP and limited expression of S100 and OLIG2 were detected in the NHA/HRas^V12^/TRIM24 S767/768A‐derived tumor xenografts (Figure S6E, Supporting Information). Taken together, our data demonstrate that DNA‐PKcs phosphorylates TRIM24 at S767/768 to promote TRIM24‐driven Ep‐GBM‐like transformation.

### 2.6 HRas^V12^ Activates PHAX to Upregulate U3 snoRNA and Thereby Facilitates PHAX Recruitment and Ku‐Dependent DNA‐PKcs Phosphorylation of TRIM24

**Figure 6:**
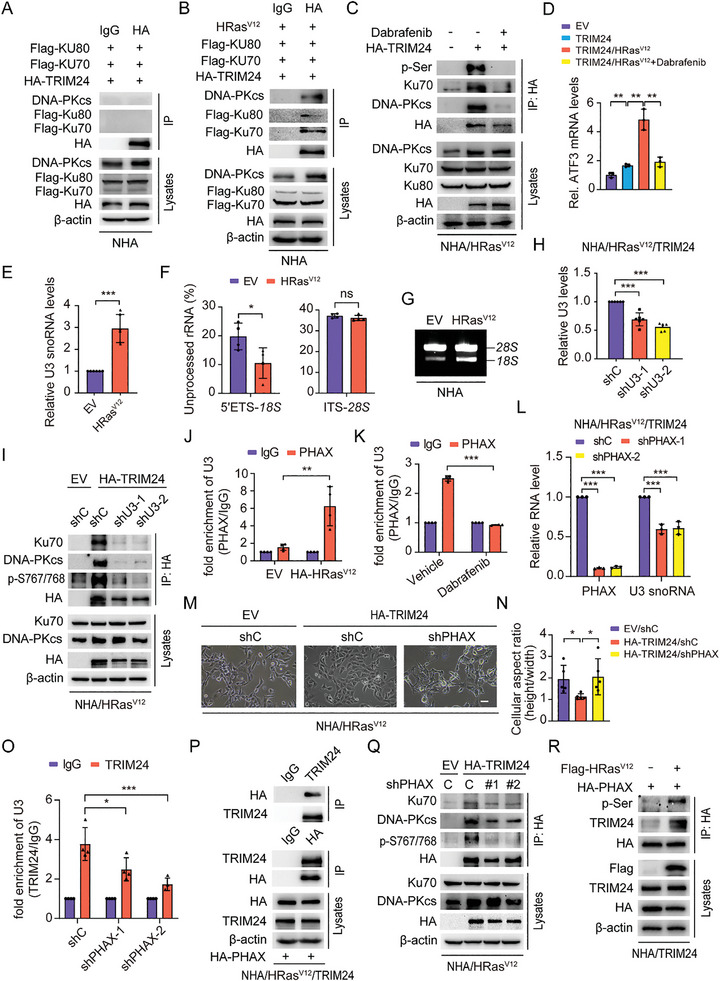
HRas^V12^ activates PHAX to upregulate U3 snoRNA and thereby facilitates PHAX recruitment and Ku‐dependent DNA‐PKcs phosphorylation of TRIM24. A,B) Co‐IP of TRIM24 interaction with the DNA‐PK complex in NHA cells without (A) or with HRas^V12^ (B). TRIM24, Ku70, and Ku80 constructs with or without HRas^V12^ were co‐transfected into NHA cells. C) Inhibitory effects of Dabrafenib (40 µm) on TRIM24 phosphorylation and protein interaction between TRIM24 and the DNA‐PK complex in NHA/HRas^V12^/TRIM24 cells. Cells were treated with Dabrafenib for 24 h. D) QRT‐PCR of effects of HRas^V12^ overexpression or inhibition by Dabrafenib on TRIM24 regulating ATF3 transcription. E) QRT‐PCR of the effects of HRas^V12^ overexpression on *U3 snoRNA* expression. F) Effects of HRas^V12^ overexpression on rRNA maturation in NHA cells determined by detecting 5′ external transcribed spacer (ETS)*−18S rRNA* and internal transcribed spacer (ITS)*−28S rRNA* using QRT‐PCR assays. G) Non‐denaturing agarose gel electrophoresis of total RNA isolated from the same number of NHA cells expressing HRas^V12^ or EV. H) QRT‐PCR of *U3 snoRNA* KD in NHA/HRas^V12^/TRIM24 cells. I) Effects of *U3 snoRNA* KD on TRIM24 S767/768 phosphorylation and its interaction with the DNA‐PK complex in NHA/HRas^V12^/TRIM24 cells. J) RNA binding protein immunoprecipitation (RIP) assay and QRT‐PCR of the effects of HRas^V12^ overexpression on the interaction between the PHAX protein and U3 snoRNA in NHA cells. K) RIP assay and QRT‐PCR analysis on the effects of Dabrafenib treatment on the interaction between PHAX protein and U3 snoRNA in NHA/HRas^V12^ cells. Cells were treated with Dabrafenib (40 µm) for 24 h. L) QRT‐PCR of the effects of *PHAX* KD on U3 snoRNA expression in NHA/HRas^V12^/TRIM24 cells. M) Representative images of the effects of *PHAX* KD on NHA/HRas^V12^/TRIM24 cell morphological changes. Scale bar, 100 µm. N) Quantification of the differences in the cell aspect ratio of cells in (M). O) RIP assay and QRT‐PCR of the effects of *PHAX* KD on the interaction between TRIM24 protein and U3 snoRNA in NHA/HRas^V12^/TRIM24 cells. P) Co‐IP of TRIM24 interaction with PHAX in NHA/HRas^V12^ cells. TRIM24 and PHAX constructs were co‐transfected into NHA/HRas^V12^ cells. Q) Effects of *PHAX* KD on TRIM24 S767/768 phosphorylation and its interaction with the DNA‐PK complex in NHA/HRas^V12^/TRIM24 cells. R) Effects of HRas^V12^ overexpression on PHAX phosphorylation and protein interaction between TRIM24 and PHAX in NHA/TRIM24 cells. Data represent two or three independent experiments with similar results. Error bars, s.d. ns indicates not significant. *P < 0.05, **P < 0.01, ***P < 0.001, by two‐way Student's *t*‐test.

To investigate the role of HRas^V12^ in TRIM24‐driven Ep‐GBM‐like transformation, we next assessed the interaction between TRIM24 and DNA‐PKcs in NHA cells and found no interaction between TRIM24, DNA‐PKcs, Ku70, and Ku80 in NHA cells lacking the HRas^V12^ mutation. However, this interaction was evident in NHA/HRas^V12^ cells (**Figure** **6**A,B). Similar results were also observed in HEK293T cells (Figure S7A,B, Supporting Information). In addition, treatment with Dabrafenib, an inhibitor of the Ras downstream effector *BRAF*,^[^ ^53^ ^]^ diminished the interaction between TRIM24 and DNA‐PKcs, reduced TRIM24 phosphorylation (Figure 6C), and impaired TRIM24‐induced Ep‐GBM‐like transformation (Figure S7C, Supporting Information). We further assessed the influence of HRas^V12^ on TRIM24 regulation of *ATF3* transcription. As shown in Figure 6D, TRIM24 regulation of *ATF3* transcription was promoted by HRas^V12^ and this effect was impaired by dabrafenib treatment. These data demonstrate that HRas^V12^ is necessary for the TRIM24‐induced Ep‐GBM‐like transformation by promoting the interaction between TRIM24 and DNA‐PKcs, which also supports the transcriptional regulatory function of TRIM24.

DNA‐PKcs is recruited by the Ku complex to bind to not only DNA during DNA damage,^[^ ^54^, ^55^ ^]^ but also RNA in ribosomal RNA (rRNA) biogenesis.^[^ ^28^ ^]^ For example, the Ku complex binds to U3 small nucleolar RNA (U3 snoRNA), which is essential for *18S* rRNA processing, and drives the assembly of DNA‐PKcs on U3 snoRNAs. U3 snoRNAs activate DNA‐PKcs to promote *18S* rRNA processing.^[^ ^28^ ^]^ Thus, we hypothesized that the HRas^V12^ mutation may upregulate U3 snoRNAs and recruit the Ku complex to facilitate the DNA‐PKcs phosphorylation of TRIM24. We first assessed the effect of HRas^V12^ mutation on the levels of U3 snoRNA. Compared with the EV control, U3 snoRNA levels were significantly higher in NHA/HRas^V12^ cells (Figure 6E). In addition, treatment with the *BRAF* inhibitor dabrafenib, DNA‐PKcs inhibitors LY294002, or NU7441 decreased the expression of U3 snoRNAs (Figure S7D–F, Supporting Information). Second, we investigated the levels of mature *28S* and *18S* rRNAs in NHA cells transfected with or without the HRas^V12^ mutation. As shown in Figure 6F, the HRas^V12^ mutation increased the levels of processed *18S* rRNAs, but not *28S* rRNAs in NHA cells. Consistently, the HRas^V12^ mutation increased mature *18S* rRNA levels (Figure 6G), further suggesting that the HRas^V12^ mutation upregulates U3 snoRNAs to regulate rRNA biogenesis in NHA cells. Finally, we knocked down *U3 snoRNA* in NHA/HRas^V12^/TRIM24 cells using two different shRNAs (Figure 6H). Compared to the control, depletion of *U3 snoRNA* markedly inhibited the interaction between the DNA‐PK complex and TRIM24, along with reduced p‐S767/768 of TRIM24 (Figure 6I). These data suggest the idea that HRas^V12^ upregulates U3 snoRNA expression to recruit and activate Ku‐dependent DNA‐PKcs to phosphorylate TRIM24.

Phosphorylated adaptor for RNA export (PHAX) is a critical component of the multiprotein complex that interacts with U3 snoRNA to regulate U3 snoRNA subcellular localization.^[^ ^56^, ^57^ ^]^ PHAX is regulated by the CK2 kinase, a key downstream factor in the RAS pathway.^[^ ^58^, ^59^, ^60^ ^]^ Thus, we determined whether PHAX was important for U3 snoRNA‐regulated DNA‐PKcs phosphorylation of TRIM24. First, compared to the control, HRas^V12^ increased the interaction between PHAX and U3 snoRNAs in NHA cells (Figure 6J). The interaction between PHAX and U3 snoRNAs was diminished in NHA/HRas^V12^ cells treated with dabrafenib (Figure 6K). Second, *PHAX* was knocked down in NHA/HRas^V12^/TRIM24 cells to reveal decreasing U3 snoRNA expression, Ep‐GBM‐like transformation being markedly reduced in NHA/HRas^V12^/TRIM24 cells (Figure 6L–N), as well as TRIM24 and U3 snoRNAs interaction being markedly reduced (Figure 6O). Third, we verified that PHAX interacted with TRIM24 in NHA/HRas^V12^/TRIM24 cells (Figure 6P). Consistent with the influence of *U3 snoRNA* KD on the interaction between TRIM24 and the DNA‐PK complex, as well as TRIM24 phosphorylation, *PHAX* KD also impaired the binding of DNA‐PKcs to and phosphorylation of TRIM24 (Figure 6Q). Finally, we found that HRas^V12^ promoted PHAX serine phosphorylation (p‐S) and its binding to TRIM24 (Figure 6R). Taken together, these data demonstrate that HRas^V12^ activated PHAX to upregulate U3 snoRNAs, thereby promoting PHAX recruitment of TRIM24 and Ku‐dependent recruitment of DNA‐PKcs to U3 snoRNAs and facilitating DNA‐PKcs phosphorylation of TRIM24.

### 2.7 Inhibition of DNA‐PKcs Impairs TRIM24‐Driven Ep‐GBM‐Like Tumor Progression

**Figure 7:**
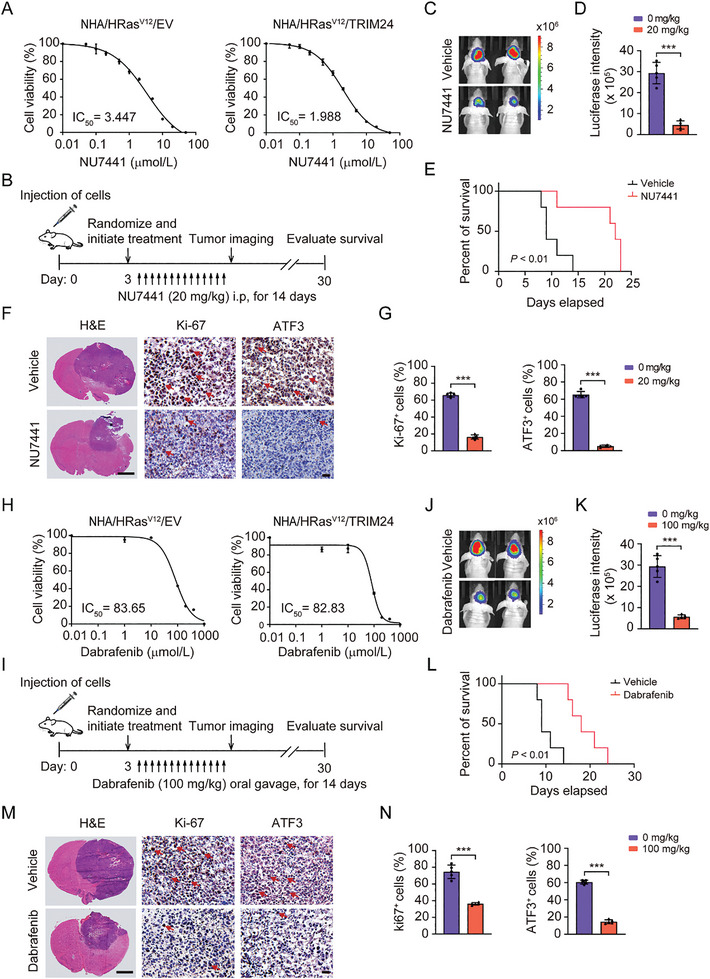
Inhibition of DNA‐PKcs impairs TRIM24‐driven Ep‐GBM progression. A) Viability of NHA/HRas^V12^ cells with or without ectopic expression of TRIM24 at 48 h after treatment with NU7441. B) Treatment scheme for the evaluation of in vivo efficacy of NU7441 in NHA/HRas^V12^/TRIM24 xenografts. The mice were treated with 20 mg kg^−1^ NU7441 for two weeks. C) Representative bioluminescent (BLI) images of treated animals on day 10. D) Quantification of the BLI activity in panels (C). E) Kaplan‐Meier survival analysis of animals with indicated NHA/HRas^V12^/TRIM24 tumors (*n* = 5). Median survival (days): vehicle (9) and NU7441(22). F) Representative images showing H&E and IHC staining of brain cross sections of NHA/HRas^V12^/TRIM24‐derived tumors from animals treated with NU7441 or vehicle for 7 days. Scale bar (H&E): 2 mm. Scale bar (IHC): 25 µm. G) Quantification of Ki‐67 and ATF3 positive cells. H) Viability of NHA/HRas^V12^ cells with or without ectopic expression of TRIM24 at 48 h after treatment with Dabrafenib. I) Treatment scheme for the evaluation of the in vivo efficacy of Dabrafenib in NHA/HRas^V12^/TRIM24 xenografts. The mice were treated with 100 mg kg^−1^ Dabrafenib for two weeks. J) Representative BLI images of treated animals on day 10. K) Quantification of the BLI activity in panels (J). L) Kaplan‐Meier survival analysis of animals with indicated NHA/HRas^V12^/TRIM24 tumors (*n* = 5). Median survival (days): vehicle (9) and Dabrafenib (19). M) Representative images showing H&E and IHC staining of brain cross sections of NHA/HRas^V12^/TRIM24‐derived tumors from animals treated with Dabrafenib or vehicle for 7 days. Scale bar (H&E): 2 mm. Scale bar (IHC): 25 µm. N) Quantitation of Ki‐67 and ATF3 positive cells. Data represent two or three independent experiments with similar results. Error bars, s.d. ***P < 0.001, by two‐way Student's *t*‐test or log‐rank analysis.

To further investigate the therapeutic vulnerability of the pharmacological inhibition of DNA‐PKcs on TRIM24‐induced Ep‐GBM‐like tumor progression, we first assessed the effects of NU7441 on NHA/HRas^V12^/TRIM24 cells. We found that, compared to NHA/HRas^V12^/EV cells, NHA/HRas^V12^/TRIM24 cells were more responsive to NU7441 (**Figure** **7**A). Furthermore, in an orthotopic intracranial NHA/HRas^V12^/TRIM24 tumor xenograft model, we treated tumor‐bearing immunodeficient mice with NU7441 via intraperitoneal injection (Figure 7B). As shown in Figure 7C–E, this treatment significantly reduced the tumor burden, as indicated by bioluminescent imaging (BLI), and markedly prolonged the overall survival of animals bearing NHA/HRas^V12^/TRIM24 brain tumor xenografts (*P *< 0.01). NU7441 treatment also decreased the protein expression of the tumor cell proliferation markers Ki‐67 and the TRIM24 downstream effector ATF3 (Figure 7F,G). These data indicate that targeting DNA‐PKcs with the inhibitor, NU7441, impairs TRIM24‐driven Ep‐GBM‐like tumor progression.

Since Dabrafenib is a clinically used agent for Ep‐GBM patients with the *BRAF V600E* mutation, we further investigated whether NU7441 treatment enhanced the anti‐tumor efficiency of Dabrafenib toward Ep‐GBM‐like tumors. As shown in Figure 7H, compared to NHA/HRas^V12^/EV cells, NHA/HRas^V12^/TRIM24 cells were not more sensitive to Dabrafenib. However, treatment with Dabrafenib significantly decreased the tumor burden (Figure 7I–K), extended the overall survival of animals bearing NHA/HRas^V12^/TRIM24 brain tumor xenografts (*P *< 0.01) (Figure 7L), and reduced tumor Ki‐67 and ATF3 protein expression (Figure 7M,N). The combination of Dabrafenib and NU7441 improved the responsiveness of NHA/HRas^V12^/TRIM24 cells to dabrafenib in vitro (Figure S8A, Supporting Information). Consistently, combination treatment with Dabrafenib and NU7441 suppressed the growth of NHA/HRas^V12^/TRIM24 cell in vitro (Figure S8B, Supporting Information). Treatment with NU7441 and Dabrafenib also significantly prolonged animal survival compared to the control (Figure S8C,D, Supporting Information). However, combination therapy did not improve the overall survival compared to monotherapy (Figure S8D, Supporting Information). These data suggest that a combination of DNA‐PKcs with NU7441 and *BRAF* (downstream of HRas^V12^) with Dabrafenib – which are in the same pathway as demonstrated in this study‐ could not yield appreciable synergistic effects in NHA/HRas^V12^/TRIM24‐driven Ep‐GBM‐like tumorigenicity. The combination of NU7441 with another clinical drug to better prolong survival warrants further investigation.

### 2.8 Targeting DNA‐PKcs with NU7441 Synergizes with Temozolomide to Reduce Ep‐GBM Tumorigenicity

**Figure 8:**
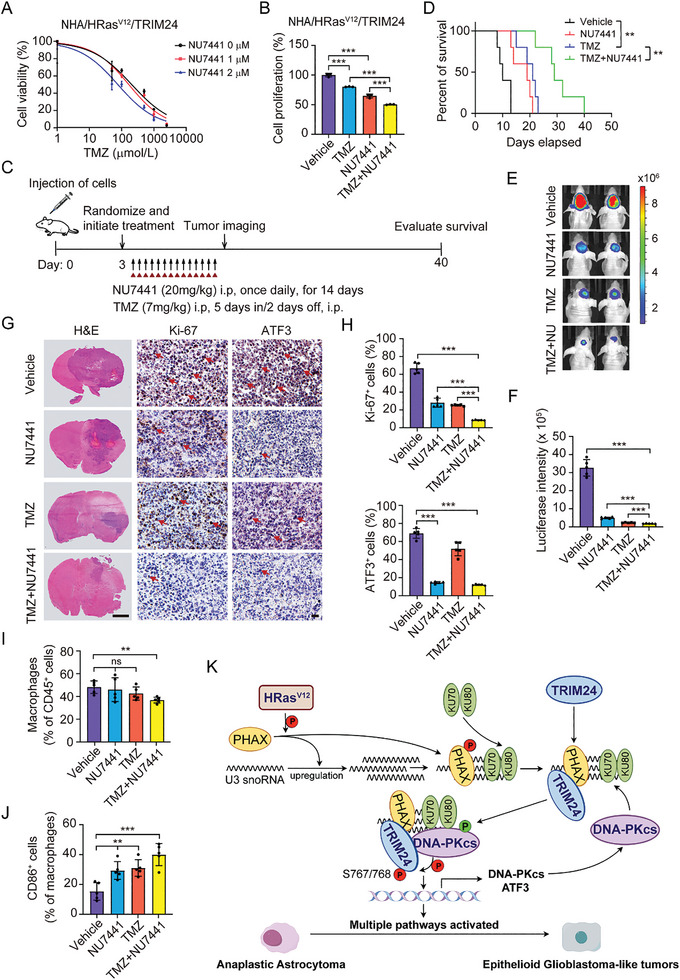
Targeting DNA‐PKcs with NU7441 synergizes with temozolomide to reduce Ep‐GBM tumorigenicity. A) Viability of NHA/HRas^V12^/TRIM24 cells at 48 h after treatment with TMZ in combination with NU7441 at indicated concentrations. B) Cell proliferation of NHA/HRas^V12^/TRIM24 cells at 72 h after treatment with NU7441 or TMZ individually or with NU7441 and TMZ combination. C) Treatment scheme for the evaluation of the in vivo efficacy of combination therapy with NU7441 and TMZ in NHA/HRas^V12^/TRIM24 xenografts. Tumor‐bearing animals were treated with the 20 mg kg^−1^ NU7441 with or without 7 mg kg^−1^ TMZ from Monday to Friday for two weeks. D) Kaplan‐Meier survival analysis of animals bearing indicated NHA/HRas^V12^/TRIM24 tumors (*n* = 5). Median survival (days): vehicle (10), NU7441(19), TMZ (21), and NU7441 + TMZ (29). E) Representative BLI images of treated mice on day 10. F) Quantification of the BLI activity in panel (E). G) Representative images of H&E and IHC staining of brain cross sections of NHA/HRas^V12^/TRIM24‐derived tumors from mice treated with TMZ or NU7441 individually or the combinations of NU7741 and TMZ for 7 days. Scale bar (H&E): 2 mm; Scale bar (IHC): 25 µm. H) Quantification of Ki‐67 and ATF3 positive cells. I,J) Tumor‐derived single cells were analyzed by flow cytometry for (I) CD11b^+^F4/80^+^ macrophages of CD45^+^ cells and (J) CD86^+^ cells of CD11b^+^F4/80^+^ macrophages. K) A working model of TRIM24‐driven glioma heterogeneity. HRas^V12^ mutation activates PHAX to upregulate U3 snoRNA levels, which further recruits Ku‐dependent DNA‐PKcs. Overexpressed TRIM24 is also recruited by PHAX to U3 snoRNAs, thereby facilitating DNA‐PKcs phosphorylation of TRIM24 at S767/768 residues. Phosphorylated TRIM24 induces epigenome and transcription factor network reprogramming and promotes glioma heterogeneity by triggering Ep‐GBM‐like transformation from AA. (Created by Figdraw). Data represent two or three independent experiments with similar results. Error bars, s.d. ns indicates not significant. **P < 0.01, ***P < 0.001, by two‐way Student's *t*‐test, one‐way ANOVA, or log‐rank analysis.

Temozolomide (TMZ) is a part of the current standard of care for patients with GBM. For patients with Ep‐GBM, TMZ treatment improves prognosis^[^ ^61^, ^62^ ^]^ but had limited effects on the progression‐free survival for these patients. We then investigated whether targeting DNA‐PKcs with NU7441 synergized with TMZ to reduce Ep‐GBM tumorigenicity. In vitro combination treatment with TMZ and NU7441 significantly enhanced the responsiveness of NHA/HRas^V12^/TRIM24 cells to TMZ in a dose‐dependent manner (**Figure** **8**A). Consistently, NU7441 synergized with TMZ to reduce NHA/HRas^V12^/TRIM24 cell growth in vitro (Figure 8B). In our orthotopic intracranial xenograft model, compared with the vehicle control or monotherapies, combination treatment with NU7441 and TMZ markedly suppressed the growth of tumor xenografts and prolonged the overall survival of tumor‐bearing animals (Figure 8C,D). Combination therapy with NU7441 and TMZ showed better therapeutic effects in inhibiting Ep‐GBM‐like tumorigenicity than monotherapies or controls (Figure 8E–H). In addition, NU7441 combined with TMZ increased the population of M1‐like CD86^+^F4/80^+^ macrophages in tumors (Figure 8I,J). Taken together, our data demonstrated that targeting DNA‐PKcs with the small‐molecule inhibitor NU7441 synergizes with TMZ to reduce Ep‐GBM tumorigenicity and prolong animal survival.

## 3 Discussion

In this study, we identified the epigenetic regulator TRIM24, which cooperates with HRas^V12^ to regulate glioma progression. Ectopic expression of TRIM24 promoted Ep‐GBM‐like transformation from HRas^V12^ AA through HRas^V12^‐activated PHAX and Ku‐dependent DNA‐PKcs (Figure 8K). Furthermore, we revealed that targeting DNA‐PKcs with the small‐molecule inhibitor NU7441 synergizes with TMZ to treat Ep‐GBM‐like tumors.

As a newly defined subtype of GBM in 2016, Ep‐GBM remains rare in clinical diagnosis, and little is known about its molecular features and biological behavior. Cases have been reported to occasionally arise from pre‐existing pleomorphic xanthoastrocytomas (PXA) with the *BRAF V600E* mutation.^[^ ^63^ ^]^ p*TERT* mutations are common in Ep‐GBM as well, along with *CDKN2A* homozygous deletion and *PDGFRA* amplification.^[^ ^32^ ^]^ Although these molecular features have been identified to characterize Ep‐GBM, the challenge remains that their existence is irregular and probabilistic, leading to difficulties in targeting unified molecules to improve the survival of most patients with Ep‐GBM. Moreover, these genetic alterations do not explain the exact molecular mechanisms underlying Ep‐GBM progression and malignancy. Although *RAS* genes are infrequently mutated in human GBM,^[^ ^64^, ^65^ ^]^ they are highly expressed in glioma tissues^[^ ^66^ ^]^ or activated in *NF1*‐loss GBMs,^[^ ^14^ ^]^ leading to frequent activation of RAS pathways. Among the oncogenes of the RAS family, when HRas^V12^ is overexpressed with activated YAP or with *p53* knockdown, both can induce mesenchymal‐like GBM in animal models.^[^ ^13^, ^14^ ^]^ Here, to our knowledge, we are the first to report that high expression of TRIM24 combined with the HRas^V12^ mutation contributes to Ep‐GBM formation, where TRIM24 phosphorylation by activated DNA‐PKcs is the critical link in this process. Additionally, we also demonstrate that Ep‐GBM‐like tumors are heterogeneous and have low M1 macrophages and high N2 neutrophils infiltration, potentially leading to the lack of immunotherapy success in these patients.

HRas^V12^ mutation‐driven transformation of immortalized normal human astrocyte (NHA)‐E6/E7/hTERT cells is related to the activation of the MAPK signaling pathway, but not through canonical activation of phospho‐MAPK, and the contributing MAPK effector molecules remain unknown.^[^ ^35^ ^]^ In this study, we report a new mechanism by which HRas^V12^ upregulates U3 snoRNAs through the activation of PHAX, a key regulator of U3 snoRNA production and subcellular localization,^[^ ^57^ ^]^ to induce malignant transformation. HRas^V12^‐upregulated U3 snoRNAs accelerate *18S* rRNA processing, which is a main component of ribosomes. This observation is consistent with the fact that malignantly transformed cells require increased ribosome generation to meet their increased demand for proteins and rapid proliferation.

Our findings also demonstrate that the recruitment of the epigenetic factor TRIM24 to snoRNAs by PHAX is important for Ep‐GBM‐like transformation. TRIM24 is an epigenetic regulator of cancer progression in various cancers.^[^ ^18^, ^67^, ^68^ ^]^ We previously reported that TRIM24 functions as a transcriptional co‐activator of STAT3 in *EGFR*‐driven GBM tumorigenesis.^[^ ^22^ ^]^ In this study, we demonstrate that TRIM24 has a novel function associated with snoRNAs. Against a background of HRas^V12^ mutation and TRIM24 overexpression, activated PHAX recruits TRIM24 to U3 snoRNAs and facilitates TRIM24 phosphorylation by Ku‐dependent DNA‐PKcs.

Consistent with previous studies showing that DNA‐PKcs binds to U3 snoRNA to promote translation in the hematopoietic process during mouse embryonic development,^[^ ^28^ ^]^ our data presented here show that during tumor progression, DNA‐PKcs is also associated with U3 snoRNA, which is required for Ep‐GBM‐like transformation. Against the background of the HRas^V12^ mutation, activated‐PHAX upregulates U3 snoRNA and recruits DNA‐PKcs through Ku70/80, which further phosphorylates TRIM24 at S767/768 to induce transformation.

More importantly, we demonstrate that the combination of a DNA‐PKcs inhibitor and TMZ was efficient in treating the highly malignant Ep‐GBM tumor, where the median OS and PFS of patients with Ep‐GBM are ≈11 and 7 months, respectively.^[^ ^32^, ^33^, ^34^ ^]^ Thus far, a limited number of cases of Ep‐GBM treatment have been reported, with regimens including surgical resection, radiotherapy, and the *BRAF‐V600E* inhibitor alone^[^ ^69^ ^]^ or combined with various targeted inhibitors such as: CSF‐1R^[^ ^70^ ^]^ inhibitors, or MEK inhibitor,^[^ ^71^, ^72^, ^73^ ^]^ or TMZ alone^[^ ^33^ ^]^ or in combination with Tumor‐Treating Fields (TTFields).^[^ ^62^ ^]^ However, the treatment efficacy was limited with patients with Ep‐GBM achieving varying degrees of prolonged median PFS (range: 8–15 months),^[^ ^69^, ^71^, ^74^ ^]^ suggesting that more universally effective treatment regimens are an urgent unmet clinical need. In this study, we show that animals bearing HRas^V12^/TRIM24‐driven GBM tumor xenografts treated with the DNA‐PKcs inhibitor NU7441 achieved prolonged survival – almost twice that of the control group. Combination treatment of NU7441 with TMZ further improved the average survival by up to one month in tumor‐bearing animals, suggesting the therapeutic potential of combination therapy targeting DNA‐PKcs with TMZ to treat patients with Ep‐GBM.

A limitation of our study is that Ep‐GBM has a low diagnostic rate; therefore, the clinical specimens that we could collect and analyze were scarce. Although we used an orthotopic intracranial xenograft model injected with NHA/HRas^V12^/TRIM24 cells to monitor clinical Ep‐GBM‐like tumor generation and progression, it is difficult to obtain fresh clinical tumor samples from patients with Ep‐GBM to establish patient‐derived xenograft (PDX) models or generate organoids to recapitulate clinical Ep‐GBM tumors. Additional clinical specimens would be beneficial for the further investigation into this rare GBM subtype.

In summary, this study presents a novel role of the epigenetic factor TRIM24 in glioma progression and Ep‐GBM‐like transformation. This study also reveals a new function of TRIM24 in binding to snoRNAs through HRas^V12^‐activated PHAX and Ku‐dependent DNA‐PKcs. The cell and mouse models characterized here will facilitate further investigations into the pathophysiology and therapeutic effects of Ep‐GBM. The results of this study may provide a step forward to a better understanding of both GBM heterogeneity and Ep‐GBM progression, thereby advancing clinical therapeutic strategies.

## 4 Experimental Section

### Cell Lines and Cell Culture

HEK293 cells were obtained from ATCC (Manassas, VA, USA). NHA/HRas^V12^ cells were generated through serially introducing pLXSP‐puro‐E6/E7, pWZL‐blast‐hTERT, and pLSXN‐neo‐H‐Ras^V12^ retroviral constructs into NHAs by Dr. Russell O. Pieper's laboratory and was a gift from him.^[^ ^35^ ^]^ NHA cells (iCell Bioscience Inc, Shanghai, P.R.C) were a gift from the Key Laboratory of Aging and Neurological Disorder Research of the Zhejiang Province. All cell lines were maintained in 10% fetal bovine serum/Dulbecco's modified Eagle's medium (DMEM) and cultured at 37 °C and 5% CO_2_. hNSCs cells were differentiated from hESC (H1, obtained from ATCC) with PSC neural induction medium kit (Gibco) and expanded in DMEM/F‐12 (Gibco). All cell lines were recently authenticated using STR DNA fingerprinting at Shanghai Biowing Applied Biotechnology Co., Ltd (Shanghai, China), and mycoplasma infection was detected using LookOut Mycoplasma PCR Detection Kit (Sigma‐Aldrich).

### Antibodies and Reagents

The following antibodies were used in this study: anti‐β‐actin (1:1000, #66009‐1‐Ig), anti‐TRIM24 (1:1000 for WB, 1:200 for IHC, #14208‐1‐AP), anti‐CRM1 (1:2000, #66763‐1‐Ig), anti‐PHAX (1:1000, #16481‐1‐AP), anti‐OLIG2 (1:400 for IHC, #66513‐1‐Ig) antibodies (Proteintech Group); anti‐H3 (1:1000, #4499), anti‐H3K27ac (1:1000, #8173), anti‐H3K27me3 (1:1000, #9733), anti‐H3K23ac (1:1000, #14 932), anti‐H3K9ac (1:1000, #9649), anti‐H3K4me3 (1:1000, #9751), anti‐DNA‐PKcs (1:1000, #12 311), anti‐Ku70 (1:1000, #4588), anti‐Ku80 (1:1000, #2753), anti‐HA Tag (1:1000, #3724), anti‐Vimentin (1:800 for IHC, #5741), anti‐CD68 (1:800 for IHC, #97 778), anti‐CD68 (1:400 for IHC, #76 437) antibodies (Cell Signaling Technology); anti‐S100 antibody (1:50 for IHC, #sc‐53438), anti‐CD45 (1:100 for IHC, #sc‐1178) antibodies (Santa Cruz Biotechnology); anti‐ki67 antibody (1:1000 for IHC, #MA5‐14520, Invitrogen); anti‐ATF3 antibody (1:1000, #A13469, ABclonal Technology); anti‐Flag antibody (1:1000, #F1804, Sigma); anti‐phosphoserine/threonine antibody (1:1000, #612 548, BD Biosciences); anti‐γ‐H2AX antibody (1:1000, #ab26350, abcam); anti‐GFAP antibody (1:500 for IHC, #Z0334, Dako). Rabbit polyclonal anti‐phospho‐TRIM24^S767/768^ antibody was raised by a pay‐for‐service vendor through immunizing animals with synthetic phospho‐peptides corresponding to residues surrounding S767/768 of human TRIM24. The antibody was then affinity purified (Abmart Inc., Shanghai, China). Rabbit anti‐normal IgG (#30000‐0‐AP, Proteintech Group). Mouse anti‐normal IgG (#sc‐2025, Santa Cruz Biotechnology). LY294002, U0126, Rapamycin, MK2206, PIK‐75, TGX‐221, NU7441, and Dabrafenib were from MCE.

### Plasmids, Lentivirus Packaging, and Infection

Flag‐TRIM24 was a gift from Michelle Barton (Addgene plasmid # 28 138).^[^ ^75^ ^]^ TRIM24 truncated constructs were constructed as previously described.^[^ ^22^ ^]^ Then, TRIM24 and its truncations were subcloned and inserted into a lentivirus pLenti‐blast vector. Point mutations were generated using a site‐directed mutagenesis kit (Invitrogen) following the manufacturer's protocol. The primer sequences are reported in Table S2 (Supporting Information). HRas^V12^ Complementary DNAs (cDNAs) were amplified by RT‐PCR, sequenced, and then subcloned into the pLenti‐blast vector. HRas^V12^ and shTP53 was cloned into the lentiviral vector LentiCRISPR‐U6‐EF1A‐T2A‐ZsGreen to generate the corresponding expression plasmids. pEGFP‐C1‐Flag‐Ku70 and pEGFP‐C1‐Flag‐Ku80 were constructed as previously described.^[^ ^76^ ^]^ PHAX cDNA was amplified, sequenced, and subcloned into the pLenti‐blast vector. To generate the lentiviral shRNA constructs against PRKDC, AFT3, U3 snoRNA, and PHAX, the shRNA sequences were cloned into the pLKO‐puro vector respectively. The shRNA sequences are reported in Table S1 (Supporting Information). Targeted cDNAs and packaging plasmids were co‐transfected into the HEK293T cells using Hieff Trans Liposomal Transfection Reagent (40802ES08, YEASEN) following the manufacturer's instruction. 48 and 72 h after transfection, the supernatants were collected, filtered, and concentrated. Cells were infected with various viruses in the presence of polybrene (5 µg ml^−1^, Sigma‐Aldrich) and selected with puromycin or blasticidin for 48 h to obtain stable clones. WB was used to validate the protein expression.

### RNA Isolation and qRT‐PCR

Total RNA was isolated from cells with TRIzol (Invitrogen) and precipitated in ethanol. After transcribing into cDNA with the Reverse Transcription Kit (Takara) according to the manufacturer's instructions, qRT‐PCR was performed in triplicate using the Universal SYBR Green qPCR Mix (Monad Biotech) on a Real‐time PCR machine (Roche). *ACTB* was used as an internal control and Primers are reported in Table S2 (Supporting Information). To evaluate the rRNA processing, qRT‐PCR was performed and the fraction of unprocessed rRNA was calculated according to methods in Zhou's recently published work.^[^ ^77^ ^]^

### Immunoprecipitation (IP) and Western blotting (WB) Assays

WB and IP analyses were performed as previously described.^[^ ^78^ ^]^ Briefly, cells were lysed in IP lysis buffer (20 mm Tris‐HCl, pH 7.5, 150 mm NaCl, 1 mm EDTA, 2 mm Na_3_VO_4_, 5 mm NaF, 1% Triton X‐100 and protease inhibitor cocktail) at 4 °C for 30 min. The lysates were centrifuged at 4 °C, 12 000 × *g* for 20 min, and then equal amounts of cell lysates were immunoprecipitated with specific antibodies or protein A/G magnetic beads (Bio‐Rad). After being washed four times with IP lysis buffer, the immunoprecipitates were resolved in a 2× SDS lysis buffer and then the standard WB was performed. The proteomics analyses for TRIM24 binding proteins in NHA/HRas^V12^/TRIM24 cells were performed at BGI‐Tech (Shen zhen, China). NHA/HRas^V12^/TRIM24 cells were immunoprecipitated with TRIM24 antibody and the protein samples were analyzed by LC‐MS/MS. The raw data were searched against the UniProt database and MS identification of DNA‐PK complex binding to TRIM24 was shown in Table S3 (Supporting Information).

### Histology and Immunohistochemistry (IHC)

In accordance with a protocol approved by the Clinical Care and Use Committee of Ren Ji Hospital (Shanghai, China), two paraffin‐embedded sections of Ep‐GBM were collected from 2018 to 2022 at Ren Ji Hospital, paraffin‐embedded sections of non‐Ep‐GBM were collected from 2006 to 2022 at Ren Ji Hospital. The informed consent was obtained from all patients. These clinical specimens were examined and diagnosed by individual pathologists at Ren Ji Hospital. The tissue sections from paraffin‐embedded de‐identified human GBM specimens were stained with antibodies against TRIM24 (1:1100), ATF3 (1: 200). Nonspecific IgGs were used as negative controls. The stained tissues were scored by two individuals blinded to the clinical parameters. Sections of Ep‐GBM xenograft tumors from mouse brains that were embedded in Optimal cutting temperature (OCT) and were separately stained with hematoxylin and antibodies as previously described.^[^ ^79^ ^]^

### Cell Proliferation and Viability Assays

Cells were plated in triplicate wells of a 96‐well microplate (3000 cells per well). Cell proliferation analysis was performed using a CellTiter‐Glo Luminescent Cell Viability Assay (CTG) (Promega). After 24 h, cells were treated with vehicle (DMSO) or NU7441 from 0.01 to 100 µm or Dabrafenib from 0.01 to 1000 µm. After being treated for 24, 48, and 72 h, cells were tested with CTG for their viability. Half‐maximal inhibitory concentration (IC_50_) values were determined from fitted concentration‐response curves obtained from three independent experiments using GraphPad Prism 9 nonlinear regression curve fit.

### RNA‐Seq and Differentially Expressed Gene Analyses

Total RNA was extracted with TRIzol reagent (Invitrogen). RNA‐Seq was performed as previously described.^[^ ^22^ ^]^ Raw sequencing reads were mapped to the human genome (hg38) and GENCODE v38 using hisat2. Mapped reads were assigned to genes by featureCounts from Rsubread. Differentially expressed genes were calculated by DESeq2. Functional annotations were done using clusterProfiler. Motif enrichment analysis was performed by findMotifs.pl from Homer.

### Flow Cytometry

After treatment, mouse tumors were collected, washed with DPBS buffer, and digested to achieve single cells. The single‐cell suspensions were then incubated on ice with fluorescent dye‐conjugated antibodies against CD11b (1:200, #69‐0112‐80, eBioscience), CD45 (1:200, #48‐0451‐82, eBioscience), F4/80 (1:200, #14‐4801‐82, eBioscience), and CD86 (1:200, #12‐0862‐82, eBioscience) in the dark for 30 min. Flow cytometry analysis was performed using the LSRII Flow Cytometer (BD Biosciences). Data were analyzed using the FlowJo software.

### RNA Immunoprecipitation (RIP) Assay

Cells were washed with PBS, digested, and centrifuged at 4 °C, 1000 ×* g* for 5 min. The precipitates were washed with PBS twice and lysed in RIP lysis buffer (100 mm HEPES, pH 7.0, 1 m KCl, 50 mm MgCl_2_, 5% NP‐40, protease inhibitor cocktail, 1 m DTT, and RNase inhibitor). After immunoprecipitation with 5 µg anti‐HA antibody (#3724, Cell Signaling Technology) or anti‐TRIM24 (#14208‐1‐AP, Proteintech Group) and protein A/G magnetic beads (Bio‐Rad), the lysates were washed with RIP wash buffer (250 mm Tris‐HCl, pH 7.0, 750 mm NaCl, 5 mm MgCl_2_, 0.25% NP‐40) six times. The co‐precipitated RNA was purified and detected by RT‐PCR. To detect RNA signals that specifically bind to HA or TRIM24, total RNA (input controls) and normal rabbit IgG controls were simultaneously assayed.

### Single‐Cell Collection

Five Athymic nu/nu female mice aged 6–8 weeks (JieSiJie Laboratory, Shanghai, P.R.C) were used for each group. After neuropsychiatric symptoms appeared the mice were euthanized. Brain tumor and paratumor tissues were collected, washed with DPBS buffer, and immediately stored in sterile RPMI 1640 medium. Then the tissues were transferred into pre‐warmed 1640 medium containing 2 mg ml^−1^ collagenase IV (Sigma) and 20 µl ml^−1^ DNase I (sigma). The mixture was gently pipetted and digested for 20 min at 37 °C to fully release single cells. The cell suspension solution was filtered and red blood cells were lysed. After centrifugation, the pellet was resuspended in DPBS and prepared for scRNA‐seq.

### Single‐Cell RNA Sequencing and Analysis

ScRNA‐seq was performed according to the manufacturer's instructions of DNBelab C4. Cell lysis and magnetic bead mRNA capture were performed in droplets. After emulsion breakage, reverse transcription was conducted. cDNA second strand was then synthesized. cDNA and oligo products were amplified and subjected to quality detection. After construction of the oligo library, the cDNAs were subjected to fragmentation, end repair, and addition of “A” bases at the 3′‐end and then subjected to adaptor ligation. PCR amplification of cDNA products was conducted. Single‐stranded cyclized cDNA products were replicated via rolling cycle amplification. A DNA nanoball (DNB) was generated and loaded into patterned nanoarrays and sequenced through combinatorial Probe‐Anchor Synthesis (cPAS). Cell Ranger software was used to process raw data. The FASTQ files were mapped to the hg38 human reference genome and mm10 mouse reference genome to distinguish human and mouse cells.

Raw sequencing data were aligned and quantified using CellRanger. The output was converted to a Seurat object using the R Seurat package for downstream analysis. To remove low‐quality cells, the following thresholds were applied: nUMI < = 20 000, nGene > = 200, nGene < = 4000, and mitoRatio < 0.1. Then all samples were combined with the IntegrateData function and the integrated data was scaled through the function of ScaleData. Cell clustering was performed using the FindClusters and the clusters were then annotated by the expression of canonical marker genes. FindAllMarkers was used to define marker genes for each cluster. Functional enrichment analyses were performed by clusterProfiler.

### Chromatin Immunoprecipitation Sequencing (CHIP‐Seq) and Analysis

NHA/HRas^V12^ cells and NHA/HRas^V12^/TRIM24 cells were cross‐linked with formaldehyde to a final concentration of 1%. The cross‐linking reaction was stopped by adding glycine, then the cells were scraped down and centrifuged at 4 °C, 2000 ×* g* for 5 min. The precipitates were resuspended in ChIP dilution buffer (50 mm HEPES, pH 7.5, 155 mm NaCl, 1.1% Triton X‐100, 0.11% NaDeoxycholate, 1 mm EDTA, 1% SDS and protease inhibitor cocktail) and sonicated. Then the samples were immunoprecipitated with an anti‐H3K27ac antibody (#8173, Cell Signaling Technology) or the relevant non‐specific IgG (30000‐0‐AP, Proteintech Group). ChIP‐Seq library generation was performed according to standard protocols using a BioScientific DNA Sample Kit. Libraries were sequenced using Illumina HiSeqX Ten platforms. Raw sequencing reads were aligned to the Human Reference Genome (hg38) using bowtie2. Duplicates were removed with Samtools. Regular peak calling was performed using MACS3. Enhancers were called using ROSE. Motif enrichment analyses were performed by findMotifsGenome.pl from Homer.

### Tumorigenesis Studies

All animal experiments were conducted in accordance with an animal use protocol approved by the Shanghai Jiao Tong University Institutional Animal Care and Use Committee (IACUC). The approval number was RJ2022‐1003. Athymic nu/nu female mice aged 6–8 weeks (JieSiJie Laboratory, Shanghai, P.R.C) were used. Mice were randomly divided into 5–6 mice per group and cells (5 × 10^5^ in 5 µl PBS) transduced with a luciferase reporter were stereotactically implanted into the mouse brain as previously described.^[^ ^80^ ^]^ After injection of D‐luciferin, bioluminescence imaging of each mouse was performed and analyzed using the IVIS Lumina imaging station (Caliper Life Sciences). Mice were euthanized when neuropathological symptoms developed. Then the brains were removed and analyzed.

### Statistics

All co‐immunoprecipitation (co‐IP) and immunostaining were repeated three times with similar results detected. GraphPad Prism version 9.0 for Windows (GraphPad Software Inc., San Diego, CA, USA) was used to perform one‐way analysis of variance (ANOVA) with Newman‐Keuls post hoc test or an unpaired, two‐tailed Student's *t* test. Kaplan‐Meier survival analysis was carried out using log‐rank test. All data were presented as mean ± SD. A *P* value of <0.05 was considered significant.

### Study Approval

All the work related to human tissues was performed at the Shanghai Jiao Tong University under institutional review board (IRB)‐approved protocols, according to NIH guidelines. All experiments using animals were performed at the Shanghai Jiao Tong University under the Institutional Animal Care and Use Committee‐approved protocols, according to NIH guidelines. The approval number was RJ2022‐1003.

## Conflict of Interest

The authors declare no conflict of interest.

## Author Contributions

C.X., G.C., and B.Y. contributed equally to this work. H.F. and Y.L. performed conceptualization, data analyses, supervision, wrote the original draft, reviewed, and edited the draft. C.X. performed methodology, data analyses, wrote the original draft, reviewed, and edited the draft. G.C. performed methodology, data investigation, visualization. B.Y. performed methodology. Y.Y. and Y.Z. performed data investigation, visualization, reviewed, and edited the draft. S.Y.C. reviewed and edited the draft. B.S., Y.X., and M.Z. reviewed and edited the draft.

## Supporting information

Supporting Information

## Data Availability

The data that support the findings of this study are available in the supplementary material of this article.
