# Supplementary material for: TRIM24 Cooperates with Ras Mutation to Drive Glioma Progression through snoRNA Recruitment of PHAX and DNA‐PKcs
Source: Adv Sci (Weinh). 2024 Jun 3;11(29):2400023. doi: 10.1002/advs.202400023 (PMC11304257; doi:10.1002/advs.202400023)
Supplement: Supplementary file 1 — Supporting Information [file ADVS-11-2400023-s001.docx]

**Supplementary data for**

**TRIM24 cooperates with Ras mutation to drive glioma progression through snoRNA recruitment of PHAX and DNA-PKcs**

Chenxin Xu, Guoyu Chen, Bo Yu, Bowen Sun, Yingwen Zhang, Mingda Zhang, Yi Yang, Yichuan Xiao, Shi-Yuan Cheng, Yanxin Li, and Haizhong Feng

Including:

Figure S1-S8

Table S1-S3

**
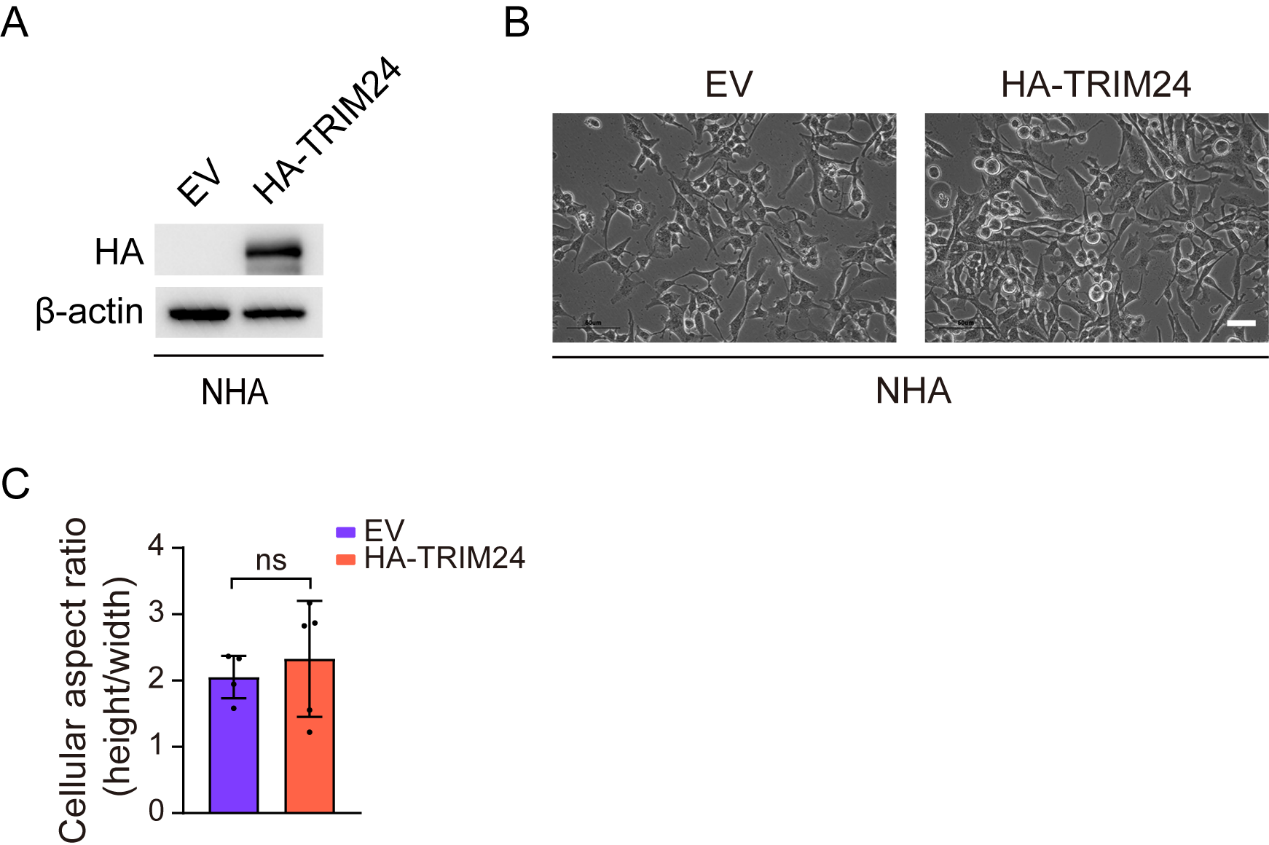
**

**Supplementary Figure 1. The influence of TRIM24 overexpression on NHA cell morphology.**

A. WB of ectopic expression of TRIM24 in NHA cells. EV, empty vector. B. Representative images of morphological changes of NHA cells transfected with TRIM24. Scale bar, 100 μm. C. Quantification of the differences in cell aspect ratio of cells in (B). Data represent two or three independent experiments with similar results. ns indicates not significant, by two-tailed Student’s t-test.

**
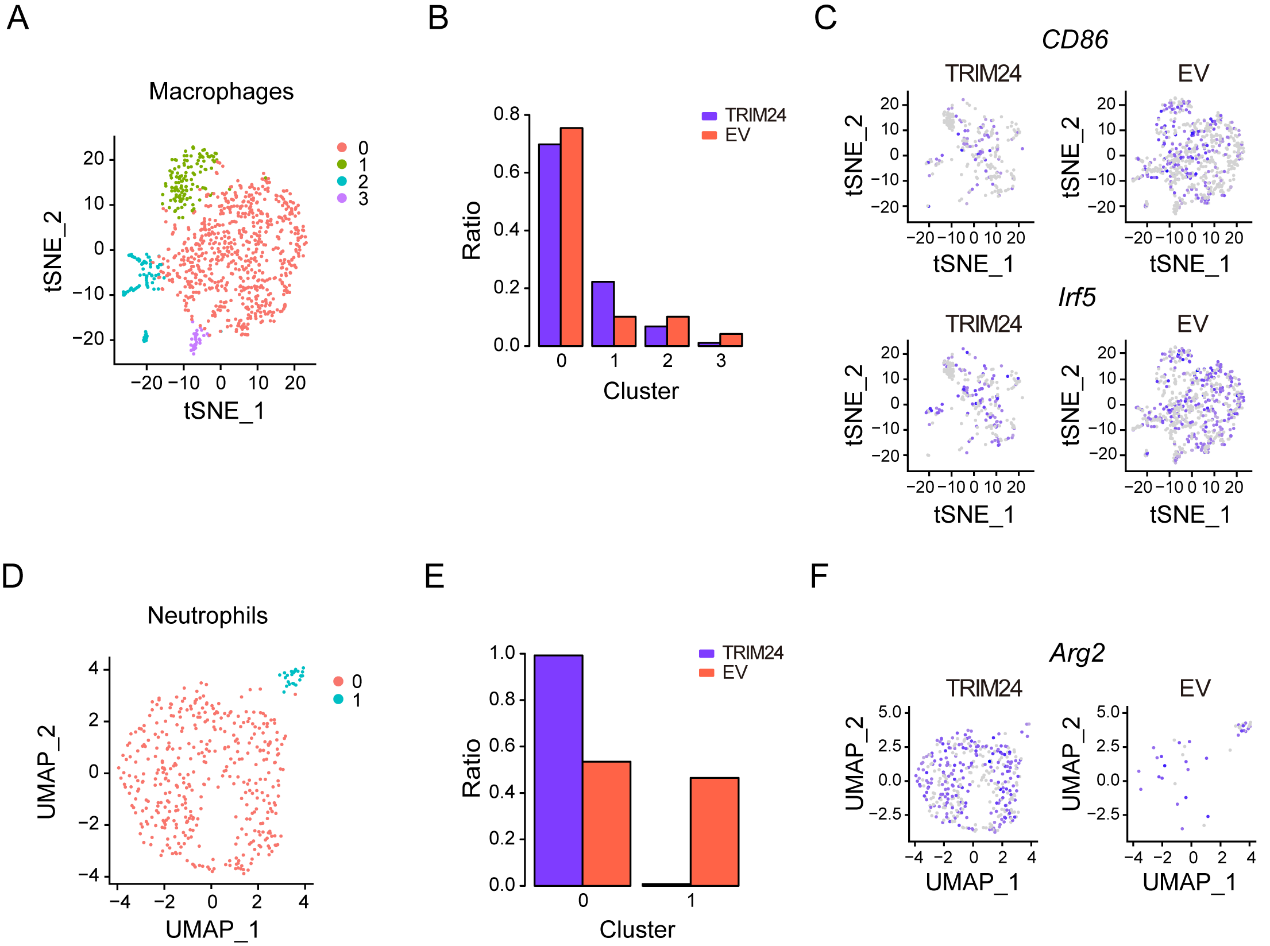
**

**Supplementary Figure 2. Polarization of infiltrated marcophages and neutrophils in TRIM24-driven Ep-GBM.**

A. tSNE (t-Distributed Stochastic Neighbor Embedding) visualizations of macrophages infiltrated in TRIM24-driven and EV-driven brain tumors. B. Composition ratio of the four clusters in TRIM24-driven and EV-driven brain tumors. C. tSNE projection of TRIM24-driven and EV-driven brain tumor cells displaying M1 macrophages markers *CD86* and *Irf5* expression. The color intensity indicates the average expression level. D. UMAP visualization of neutrophils infiltrated in TRIM24-driven and EV-driven brain tumors. E. Composition ratio of the two clusters in TRIM24-driven and EV-driven brain tumors. F. UMAP projection of TRIM24-driven and EV-driven brain tumor cells displaying N2 neutrophil marker *Arg2* expression. The color intensity indicates the average expression level.


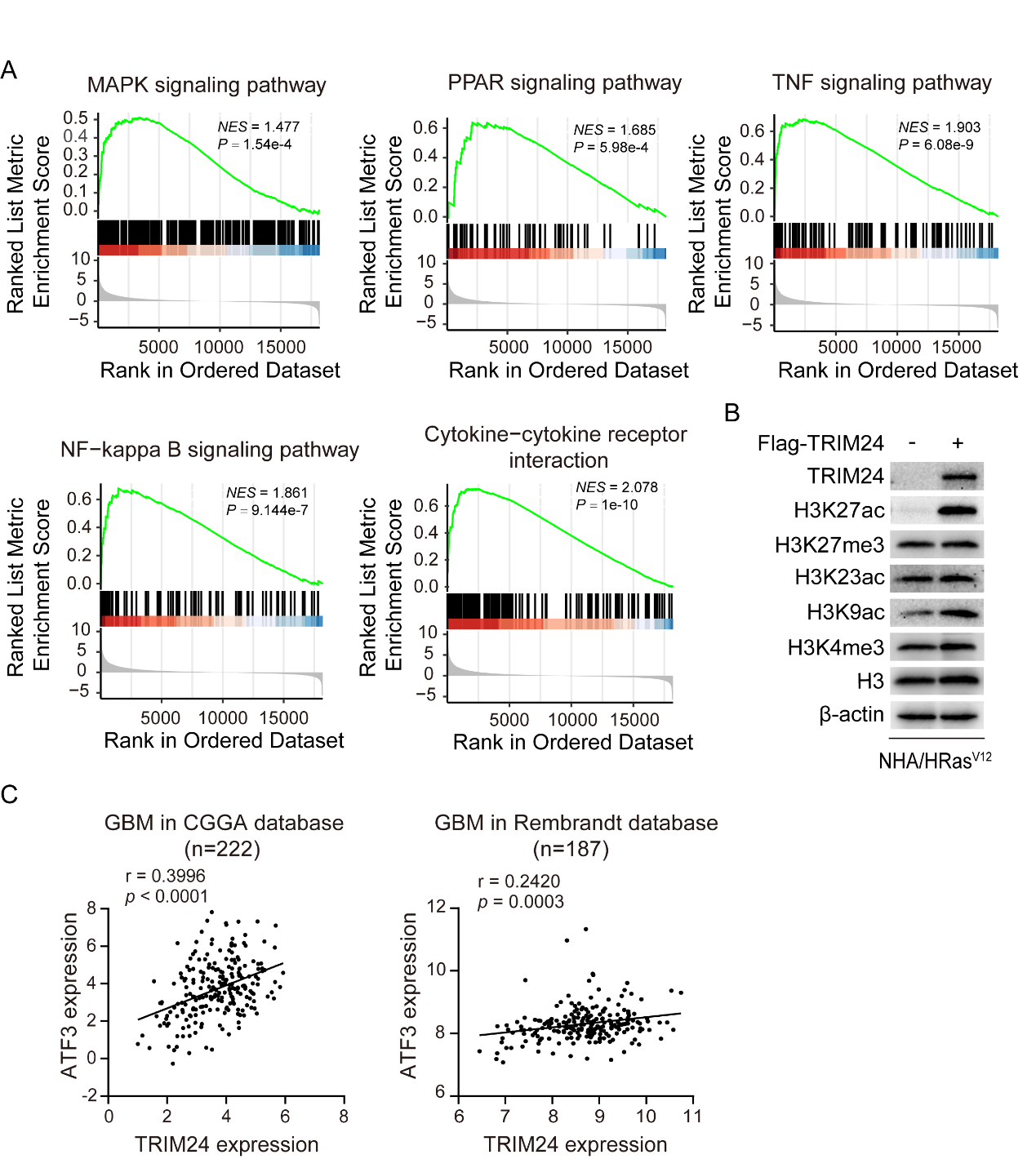


**Supplementary Figure 3. TRIM24 activates multiple signaling pathways to induce Ep-GBM-like transformation through increasing ATF3 transcription.**

A. GSEA analysis of TRIM24-activated signaling pathways using ranked gene expression changes in NHA/HRas^V12^/TRIM24 cells compared to NHA/HRas^V12^/Vector cells. *NES*, normalized enrichment score. B. WB of the effect of TRIM24 overexpression on multiple H3 modifications in NHA/HRas^V12^ cells. C. The correlation between *ATF3* expression and *TRIM24* expression in GBM patients in CGGA and Rembrandt databases.


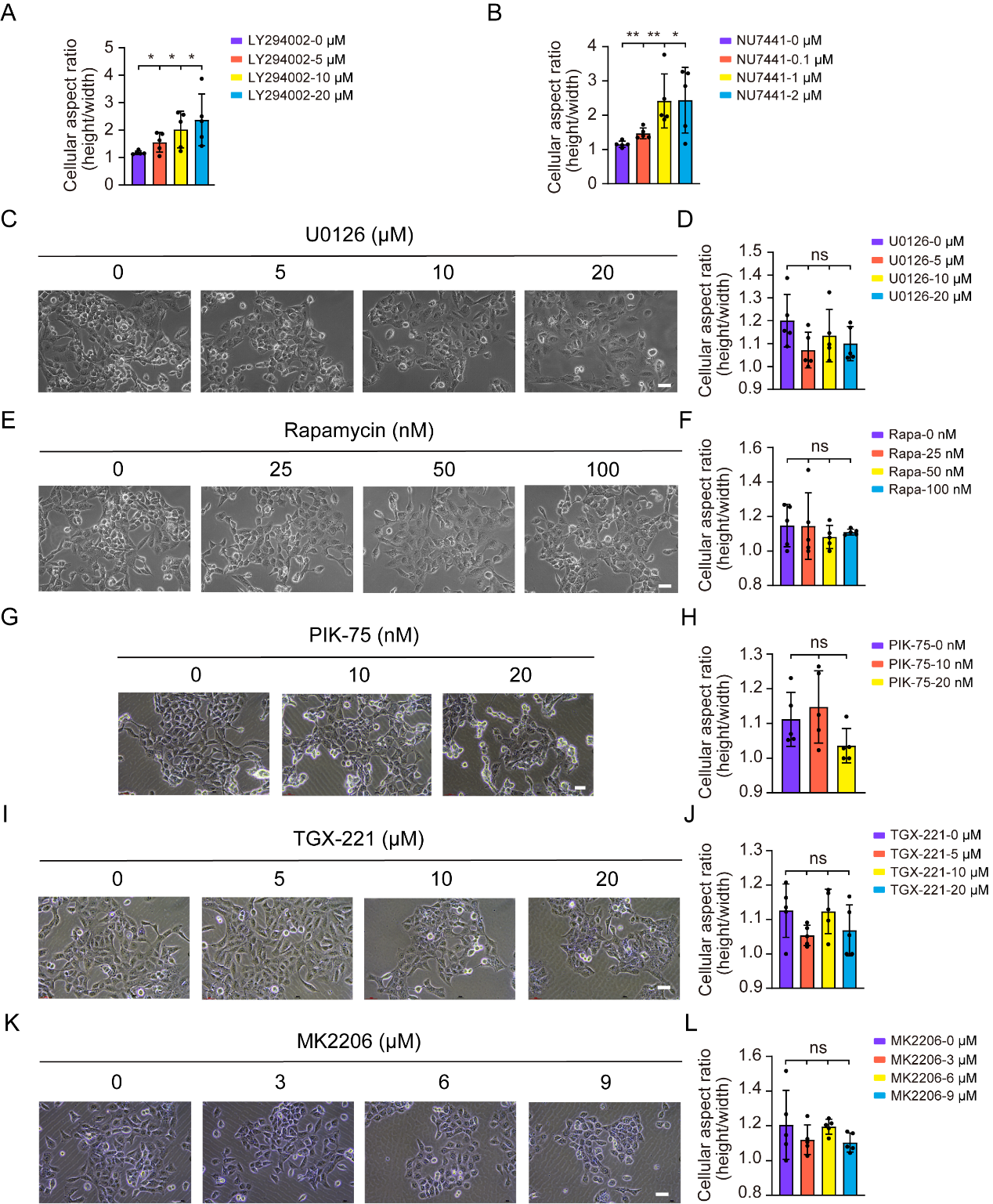


**Supplementary Figure 4.** **Effects of various inhibitors of RAS-RAF-MEK-ERK and RAS-PI3K-AKT- mTOR pathways on the morphorlogy of NHA/HRas^V12^/TRIM24 cells.**

A. Quantification of the differences in the cell aspect ratio of cells in (Figure 4A). B. Quantification of the differences in the cell aspect ratio of cells in (Figure 4C). C. Representative images of morphological changes of NHA/HRas^V12^/TRIM24 cells treated with U0126 at indicated concentrations for 24 h. Scale bar, 100 μm. D. Quantification of the differences in the cell aspect ratio of cells in (C). E. Representative images of morphological changes of NHA/HRas^V12^/TRIM24 cells treated with Rapamycin at indicated concentrations for 24 h. Scale bar, 100 μm. F. Quantification of the differences in the cell aspect ratio of cells in (E). G. Representative images of morphological changes of NHA/HRas^V12^/TRIM24 cells treated with PIK-75 at indicated concentrations for 24 h. Scale bar, 100 μm. H. Quantification of the differences in the cell aspect ratio of cells in (G). I. Representative images of morphological changes of NHA/HRas^V12^/TRIM24 cells treated with TGX-221 at indicated concentrations for 24 h. Scale bar, 100 μm. J. Quantification of the differences in the cell aspect ratio of cells in (I). K. Representative images of morphological changes of NHA/HRas^V12^/TRIM24 cells treated with MK2206 at indicated concentrations for 24 h. Scale bar, 100 μm. L. Quantification of the differences in the in cell aspect ratio of cells in (K). Data represent two or three independent experiments with similar results. ns indicates not significant. **P* < 0.05, ***P* < 0.01, by two-tailed Student’s t-test or one-way ANOVA analysis.


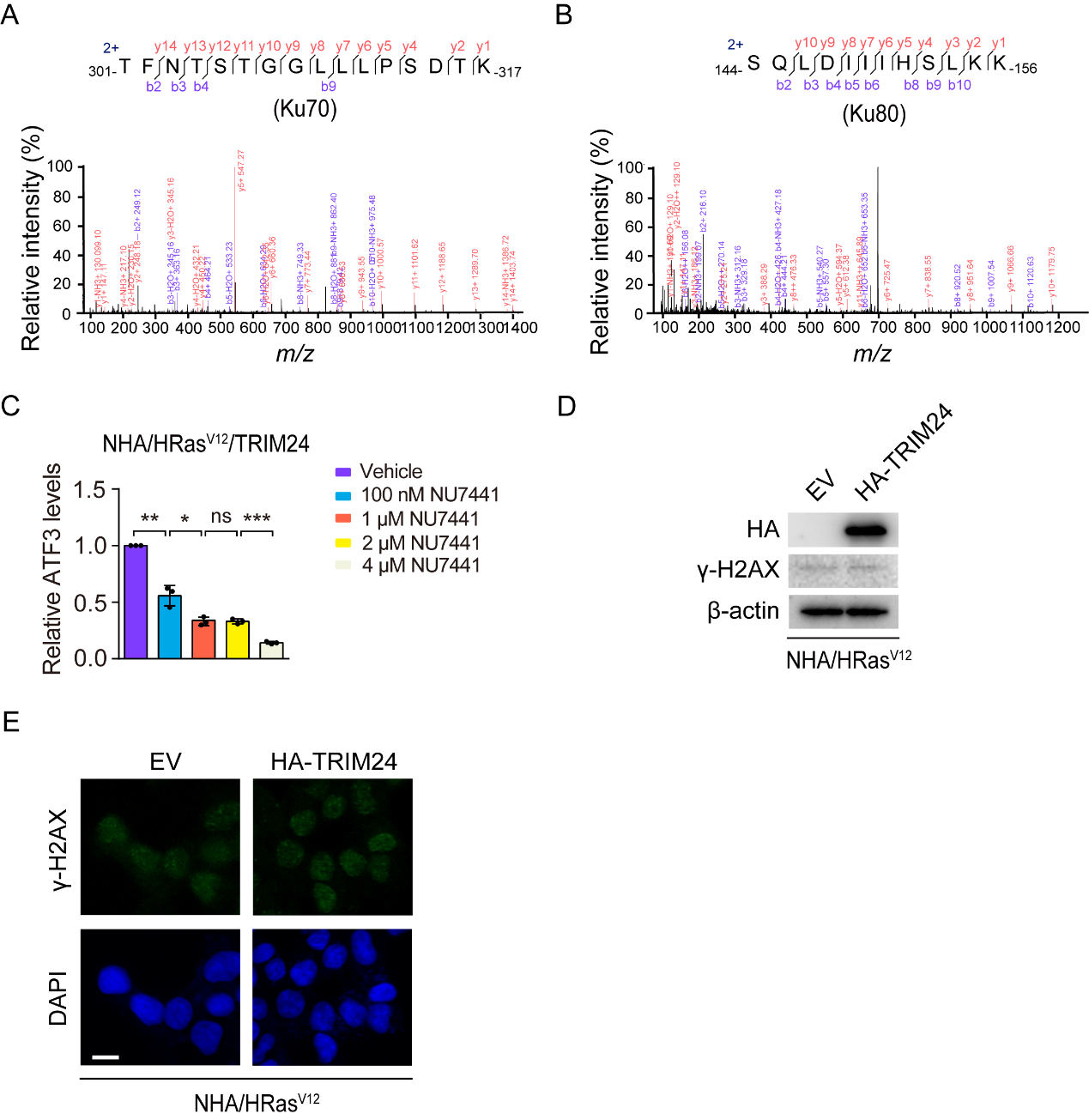


**Supplementary Figure 5.** **Identification of Ku70 and Ku80 peptides and the effect of DNA-PKcs inhibitors on ATF3 transcription, and the effect of TRIM24 overexpression on DNA damage.**

A and B. Identification of Ku70 and Ku80 peptides in the TRIM24-interacting proteins immunoprecipitated from NHA/HRas^V12^/TRIM24 cells by mass spectrometry analysis of the peptides covering parts of the protein sequences. C. QRT-PCR for the effects of NU7441 on *ATF3* transcription in NHA/HRas^V12^/TRIM24 cells. Cells were treated with NU7441 at indicated concentrations for 24 h. D. WB of the effect of TRIM24 overexpression on γ-H2AX expression in NHA/HRas^V12^ cells. E. IF analysis of γ-H2AX foci formation in NHA/HRas^V12^/EV cells and NHA/HRas^V12^/TRIM24 cells. Data represent two or three independent experiments with similar results. ns indicates not significant. **P* < 0.05, ** *P* < 0.01, ****P* < 0.001, by two-way Student’s *t*-test.


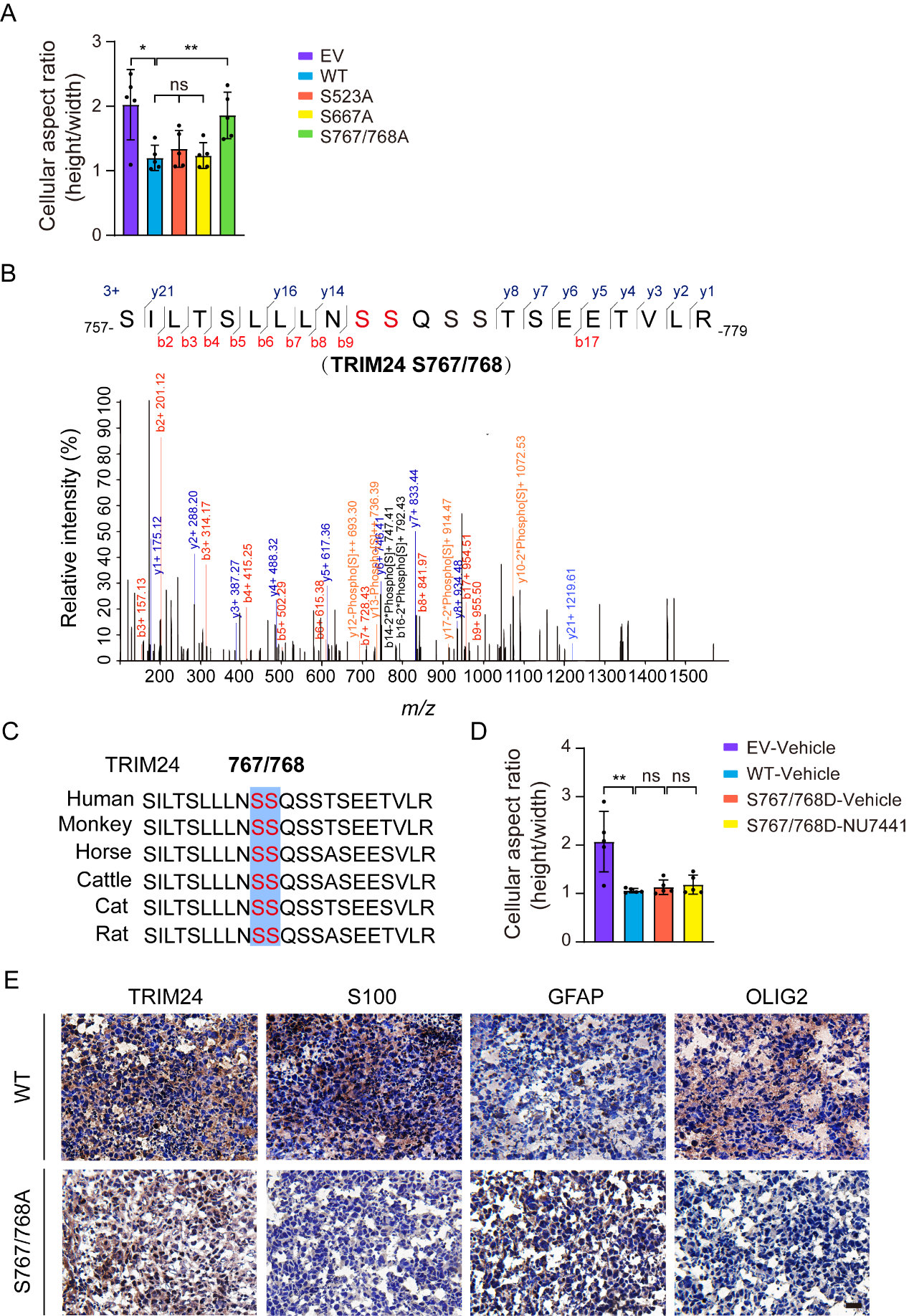


**Supplementary Figure 6.** **Identification of the phosphorylation sites of TRIM24 that promote Ep-GBM-like transformation.**

A. Quantification of the differences in the cell aspect ratio of cells in (Figure 5H). B. MS analysis of the phosphorylation site of TRIM24 by DNA-PKcs. C. The amino acid sequences around S767/768 in TRIM24 among multiple species. D. Quantification of the differences in the cell aspect ratio of cells in (Figure 5L). E. Representative images of the IHC analysis of the indicated protein in NHA/HRas^V12^/TRIM24^WT^ xenograft tumors and in NHA/HRas^V12^/TRIM24^S767/768A^ xenograft tumors. Scale bar, 25 μm. Data represent two or three independent experiments with similar results. ns indicates not significant. **P* < 0.05, ***P* < 0.01, by two-tailed Student’s t-test or one-way ANOVA analysis.


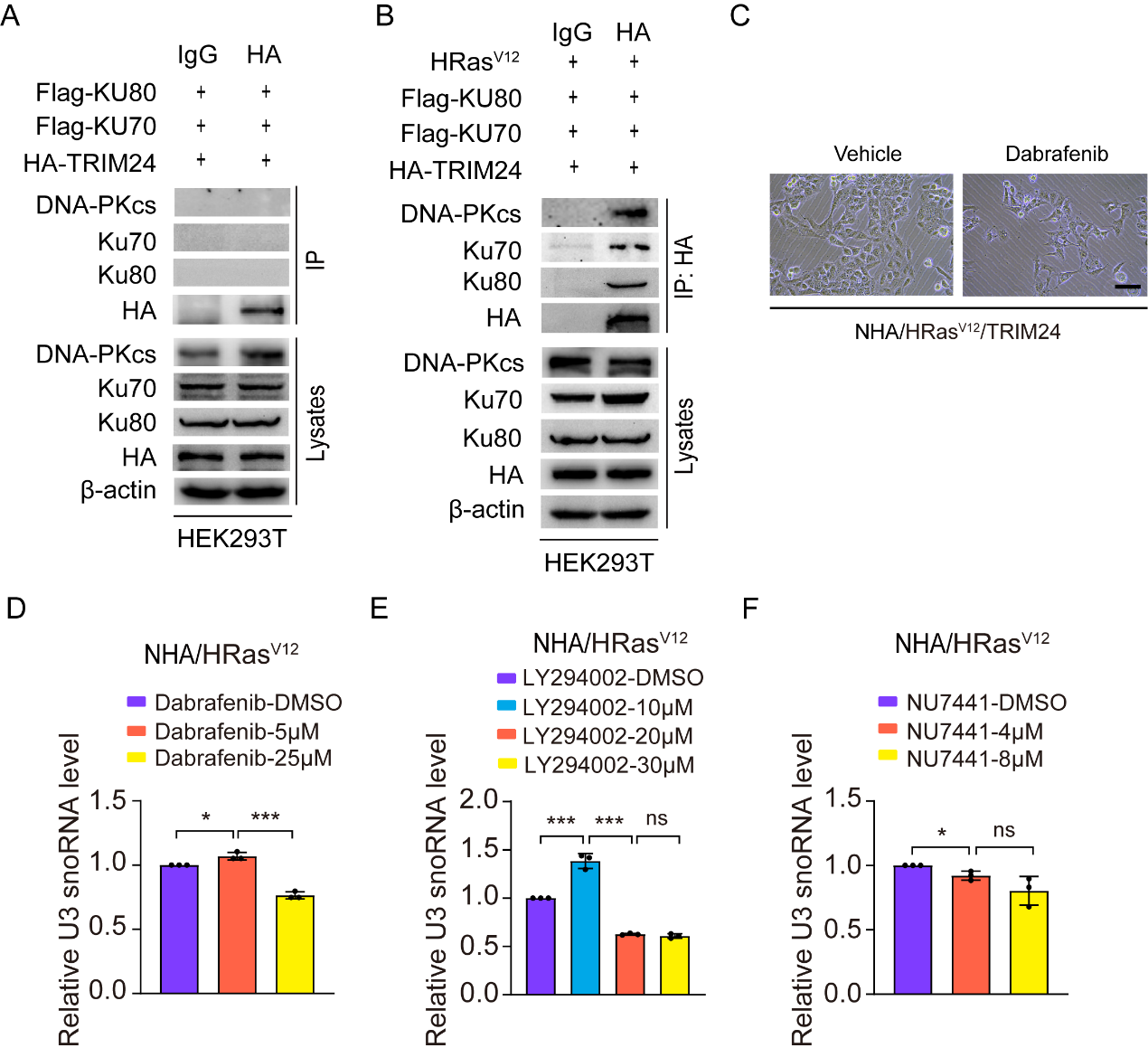


**Supplementary Figure 7.** **HRas^V12^ is required for the association between DNA-PKcs and TRIM24 and increases U3 snoRNA expression.**

A. Co-IP analyses of protein interactions between TRIM24 and the DNA-PK complex in 293T cells. TRIM24, Ku70, and Ku80 constructs were co-transfected into 293T cells. B. Co-IP analyses of protein interactions between TRIM24 and the DNA-PK complex in 293T cells. TRIM24, Ku70, Ku80, and HRas^V12^ constructs were co-transfected into 293T cells. C. Representative images of morphological changes of NHA/HRas^V12^/TRIM24 cells treated with Dabrafenib (40 μM) for 24 h. Scale bar, 100 μm. D-F. QRT-PCR analysis of the inhibitory effects of Dabrafenib (D), LY294002 (E), or NU7441 (F) on U3 snoRNA expression in NHA/HRas^V12^ cells. Cells were treated with Dabrafenib , LY294002 or NU7441 at indicated concentrations for 24 h. Data represent two or three independent experiments with similar results. ns indicates not significant. **P* < 0.05, ****P* < 0.001, by two-way Student’s *t*-test.

**
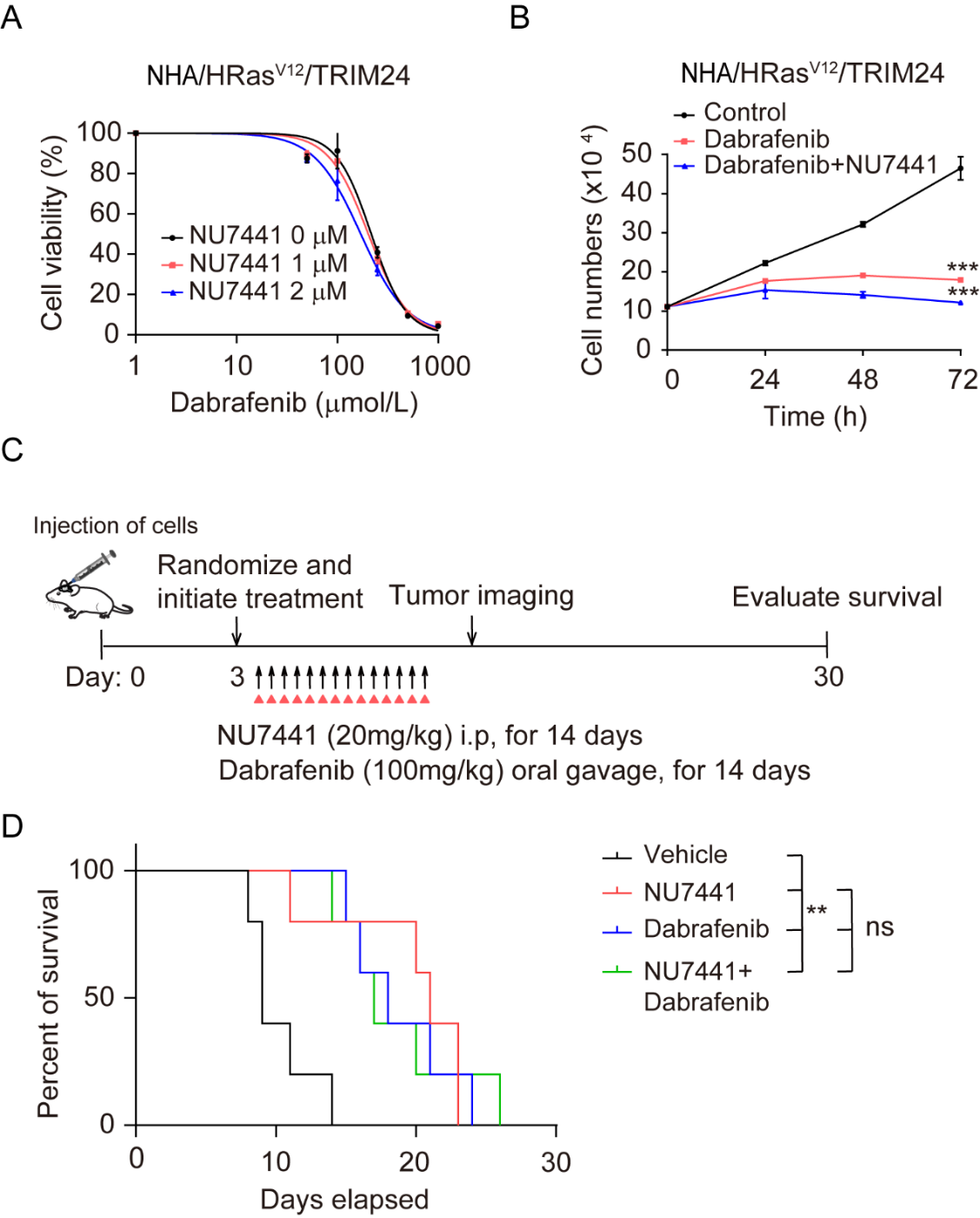
**

**Supplementary Figure 8.** **Combination treatment of both DNA-PKcs and *BRAF-V600E* does not improve Ep-GBM treatment compared with monotherapy.**

A. Viability of NHA/HRas^V12^/TRIM24 cells at 48 h after treatment with Dabrafenib combined with various concentrations of NU7441. B. Effect of Dabrafenib monotherapy or Dabrafenib combined with NU7441 on NHA/HRas^V12^/TRIM24 cell proliferation. C. Treatment scheme for the evaluation of in vivo efficacy of Dabrafenib combined with NU7441 in NHA/HRas^V12^/TRIM24 xenografts. The mice were treated with 20 mg kg^-1^ NU7441 plus 100 mg kg^-1^ Dabrafenib for two weeks. D. Kaplan-Meier survival analysis (n = 5). Median survival (days): vehicle (9), NU7441(22), Dabrafenib (18), and NU7441+ Dabrafenib (17). Data represent two or three independent experiments with similar results. Error bars, s.d. ns indicates not significant. ***P* < 0.01, ****P* < 0.001, by two-tailed Student’s t-test and log-rank analysis.

**Supplementary Table 1. Sequences of shRNAs constructed in this study.**

| shATF3-1 | GCTGAACTGAAGGCTCAGATT |
| --- | --- |
| shATF3-2 | ACGAGAAGCAGCATTTGATAT |
| shPRKDC-1 | CAGTATTGAATTTCGTGAATG |
| shPRKDC-2 | GCACCTTACTCTGTTGAAATT |
| shPRKDC-3 | CAAGCGACTTTATAGCCTT |
| shU3-1 | GTGTGTTACTAGAGAAGTTTC |
| shU3-2 | CTAGAGAAGTTTCTCTGAACG |
| shPHAX-1 | GCCCGAGTAGTGAGGATTATT |
| shPHAX-2 | CAGATGATGATAGCTGTCTTT |
| shTP53 | GTCCAGATGAAGCTCCCAGAA |

**Supplementary Table 2. Primers for mutagenesis, QRT-PCR, and RIP-qPCR assays.**

| **Mutagenesis primers** | **forward** | **reverse** |
| --- | --- | --- |
| TRIM24-S767/768A | TGCTCTTAAATGCCGCTCAGAGCTCTAC | GTAGAGCTCTGAGCGGCATTTAAGAGCA |
| TRIM24-S767/768D | TGCTCTTAAATGACGATCAGAGCTCTAC | GTAGAGCTCTGATCGTCATTTAAGAGCA |
| TRIM24-S523A | TTCAGAATCACGCCCCCAAACCCAA | TTGGGTTTGGGGGCGTGATTCTGAA |
| TRIM24-S667A | CATCAGTGCCAGCTCCAGGCCTTGC | GCAAGGCCTGGAGCTGGCACTGATG |
|  |  |  |
| **QRT-PCR and RIP-PCR primers** | **forward** | **reverse** |
| TRIM24 | GCACCTGTGGGTTTACCAA | ACTGCTGATCTGCCACTGTTT |
| PRKDC | GACGGACCTACTACGACTG | AGACAAAGGGTGGAAAGA |
| ATF3 | AGTGCTTCTGCCATCGTC | TTCTTTCTCGTCGCCTCT |
| U3 | CGTGTAGAGCACCGAAAACC | CGCTCAGGAGAAAACGCTAC |
| PHAX | GCGACAGTGCTATGAGGG | GGTTTGGGAGGAGGGTTA |
| Total 18s rRNA | GCGGGACACTCAGCTAAGAGC | GGCCCTGTAATTGGAATGAG |
| Total 28s rRNA | CTTACGGTACTTGTTGACTATCGGTCTCG | CCAAGTCCTTCTGATCGAGGCCC |
| Unprocessed-18s rRNA | GCGCCCGTCGGCATGTATTAGCTC | CTCGCCGCGCTCTACCTTACCTACCTGG |
| Unprocessed-anti-18s rRNA | GGCAGGATCAACCAGGTAGGTAAGG | GGGGGGCGGGTGGTTGGGGCGTCC |
| Unprocessed-28s rRNA | CGCTGGGCTCTTCCCTGTTCACTCG | CCCGTCCCCCTCCGAGACGCGACC |
| Unprocessed-anti-28s rRNA | CGTCTGATCTGAGGTCGCGTCTCGG | CTCTCTCCCGTCGCCTCTCCCC |
| ACTB  (normalization) | CATGTACGTTGCTATCCAGGC | CTCCTTAATGTCACGCACGAT |

**Supplementary Table 3. Mass spectrometry identification of DNA-PK complex binding to TRIM24 in NHA/HRas^V12^/TRIM24 cells.**

| **prot_acc** | **Theo. MH+[Da]** | **qvality pep** | **Ions Score** | **percolator pep** | **pep_seq** |
| --- | --- | --- | --- | --- | --- |
| P78527 | 1338.716 | 2.95E-05 | 75 | 1.11E-05 | LGASLAFNNIYR |
| P78527 | 1574.802 | 4.46E-06 | 77 | 1.43E-06 | NLSSNEAISLEEIR |
| P78527 | 1619.81 | 0.00051 | 52 | 0.000247 | FYQGFLFSEKPEK |
| P78527 | 1420.699 | 4.38E-05 | 44 | 1.71E-05 | DLLLNTMSQEEK |
| P78527 | 2400.208 | 3.81E-09 | 79 | 6.5E-10 | IIANALSSEPACLAEIEEDKAR |
| P78527 | 1218.709 | 4.21E-05 | 76 | 1.65E-05 | LLALNSLYSPK |
| P78527 | 2121.101 | 0.000485 | 28 | 0.000234 | LAGANPAVITCDELLLGHEK |
| P78527 | 1520.854 | 0.000335 | 50 | 0.000157 | HSSLITPLQAVAQR |
| P78527 | 2315.009 | 0.000104 | 48 | 4.36E-05 | LTPLPEDNSMNVDQDGDPSD  R |
| P78527 | 1210.694 | 0.000855 | 38 | 0.000436 | LATTILQHWK |
| P78527 | 1602.831 | 0.001983 | 20 | 0.001085 | MSPYKDILETHLR |
| P78527 | 1448.741 | 2.24E-05 | 76 | 8.24E-06 | SQGCSEQVLTVLK |
| P78527 | 1311.65 | 0.000154 | 55 | 6.71E-05 | YPEETLSLMTK |
| P78527 | 1678.916 | 0.000343 | 33 | 0.000162 | LVINTEEVFRPYAK |
| P78527 | 1443.799 | 0.003156 | 27 | 0.001801 | VVQMLGSLGGQINK |
| P78527 | 1894.875 | 0.00206 | 20 | 0.001138 | EQQHVMEELFQSSFR |
| P78527 | 1600.942 | 0.000143 | 56 | 6.22E-05 | NLLIFENLIDLKR |
| P78527 | 3142.69 | 3.1E-05 | 30 | 1.18E-05 | AGLLHNILPSQSTDLHHSVGT  ELLSLVYK |
| P78527 | 2177.189 | 0.000554 | 18 | 0.000272 | LLQIIERYPEETLSLMTK |
| P78527 | 1149.651 | 0.004536 | 60 | 0.002672 | AALSALESFLK |
| P78527 | 2477.251 | 2.95E-08 | 54 | 6.05E-09 | SHFASSHALICISHWILGIGDR |
| P78527 | 2076.083 | 6.63E-08 | 58 | 1.46E-08 | AVAFFLESIAMHDIIAAEK |
| P78527 | 2857.455 | 3.71E-06 | 31 | 1.16E-06 | VYELLGLLGEVHPSEMINNAE  NLFR |
| P78527 | 1879.069 | 8.39E-06 | 67 | 2.84E-06 | YKEVYAAAAEVLGLILR |
| P78527 | 1904.038 | 2.93E-06 | 92 | 9.02E-07 | LLLQGEADQSLLTFIDK |
| P78527 | 1069.575 | 0.006352 | 39 | 0.003864 | FLCIFLEK |
| P78527 | 2295.165 | 6.98E-07 | 48 | 1.88E-07 | STVLTPMFVETQASQGTLQTR |
| P78527 | 1037.603 | 0.005849 | 32 | 0.003547 | LPLISGFYK |
| P78527 | 2381.219 | 7.98E-08 | 64 | 1.78E-08 | ILELSGSSSEDSEKVIAGLYQR |
| P78527 | 1238.66 | 0.003771 | 31 | 0.002184 | AYVPALQMAFK |
| P78527 | 1322.677 | 0.003096 | 42 | 0.001762 | MSTSPEAFLALR |
| P78527 | 1815.022 | 3.1E-05 | 35 | 0.000655 | TVGALQVLGTEAQSSLLK |
| P78527 | 3234.523 | 0.000122 | 17 | 5.24E-05 | SSFDWLTGSSTDPLVDHTSPS  SDSLLFAHK |
| P78527 | 1636.825 | 1.11E-05 | 69 | 3.84E-06 | SDPGLLTNTMDVFVK |
| P78527 | 1234.669 | 0.012515 | 27 | 0.008124 | QFINLMLPMK |
| P78527 | 1620.824 | 2.99E-05 | 43 | 1.13E-05 | ETGLMYSIMVHALR |
| P78527 | 1917.905 | 1.03E-09 | 96 | 1.56E-10 | ATQQQHDFTLTQTADGR |
| P78527 | 1176.568 | 0.001222 | 35 | 0.00064 | LACDVDQVTR |
| P78527 | 1103.569 | 0.002558 | 60 | 0.001437 | LQETLSAADR |
| P78527 | 1107.688 | 0.004283 | 34 | 0.002518 | LLPAELPAKR |
| P78527 | 1480.701 | 0.000675 | 44 | 0.000337 | ILELSGSSSEDSEK |
| P78527 | 1383.748 | 0.001745 | 26 | 0.000946 | LGLPGDEVDNKVK |
| P78527 | 1467.707 | 2.65E-06 | 68 | 8.08E-07 | SLGPPQGEEDSVPR |
| P78527 | 1326.749 | 0.000237 | 50 | 0.000108 | QGNLSSQVPLKR |
| P78527 | 1175.627 | 0.002726 | 59 | 0.001543 | VTELALTASDR |
| P78527 | 2046.991 | 1.22E-12 | 103 | 1.02E-13 | INQVFHGSCITEGNELTK |
| P78527 | 1591.823 | 0.004338 | 39 | 0.002546 | LLNTWTNRYPDAK |
| P78527 | 1300.722 | 6.93E-05 | 68 | 2.82E-05 | QITQSALLAEAR |
| P78527 | 1396.695 | 1.84E-05 | 68 | 6.68E-06 | LNESTFDTQITK |
| P78527 | 1390.66 | 0.007304 | 31 | 0.004493 | NELEIPGQYDGR |
| P78527 | 929.5852 | 0.018043 | 44 | 0.01207 | MAVLALLAK |
| P78527 | 1159.535 | 0.01522 | 46 | 0.01002 | DLCNTHLMR |
| P78527 | 995.6499 | 0.016835 | 41 | 0.0112 | IPALDLLIK |
| P78527 | 1205.736 | 0.01438 | 18 | 0.009442 | LGNPIVPLNIR |
| P78527 | 951.5873 | 0.026786 | 47 | 0.01865 | LLPAELPAK |
| P78527 | 996.5473 | 0.04374 | 38 | 0.03182 | DILETHLR |
| P78527 | 933.5152 | 0.049764 | 52 | 0.03678 | YAVPSAGLR |
| P78527 | 1135.611 | 0.05868 | 29 | 0.04389 | HGDLPDIQIK |
| P78527 | 956.5775 | 0.089332 | 36 | 0.06974 | LLEEALLR |
| P78527 | 1373.758 | 0.093103 | 14 | 0.07308 | LYSLALHPNAFK |
| P78527 | 1293.68 | 0.118947 | 25 | 0.09565 | DQNILLGTTYR |
| P78527 | 1496.8 | 0.126705 | 5 | 0.1025 | NCISTVVHQGLIR |
| P78527 | 878.5168 | 0.149346 | 30 | 0.1231 | LLNFLMK |
| P78527 | 1053.642 | 0.149346 | 19 | 0.1229 | AIRPQIDLK |
| P78527 | 905.4839 | 0.184262 | 23 | 0.1545 | LLQDFNR |
| P78527 | 1009.572 | 0.194521 | 24 | 0.164 | EIFNFVLK |
| P13010 | 1394.837 | 0.00525 | 24 | 0.003143 | SQLDIIIHSLKK |
| P13010 | 1266.742 | 0.000642 | 44 | 0.000318 | SQLDIIIHSLK |
| P13010 | 1317.68 | 0.004422 | 46 | 0.002607 | HIEIFTDLSSR |
| P13010 | 1112.562 | 0.001344 | 45 | 0.00071 | YGSDIVPFSK |
| P13010 | 1880.949 | 2.23E-05 | 23 | 0.04595 | KDEKTDTLEDLFPTTK |
| P13010 | 4061.984 | 7.3E-13 | 44 | 5.81E-14 | QVFAENKDEIALVLFGTDGTD  NPLSGGDQYQNITVHR |
| P13010 | 2901.346 | 8.89E-07 | 34 | 2.46E-07 | ETVYCLNDDDETEVLKEDIIQ  GFR |
| P13010 | 3069.556 | 7.67E-05 | 6 | 3.15E-05 | IQPGSQQADFLDALIVSMDVI  QHETIGK |
| P13010 | 2321.169 | 3.13E-12 | 85 | 2.83E-13 | YAPTEAQLNAVDALIDSMSLA  K |
| P13010 | 3181.656 | 9.79E-09 | 46 | 1.81E-09 | IQPGSQQADFLDALIVSMDVI  QHETIGKK |
| P13010 | 2254.223 | 7.16E-07 | 57 | 1.94E-07 | QLNHFWEIVVQDGITLITK |
| P13010 | 1914.952 | 6.72E-08 | 56 | 3.17E-07 | HLMLPDFDLLEDIESK |
| P13010 | 1930.947 | 9.41E-05 | 50 | 3.94E-05 | HLMLPDFDLLEDIESK |
| P13010 | 1380.689 | 2.1E-05 | 57 | 7.68E-06 | TDTLEDLFPTTK |
| P13010 | 3121.482 | 2.41E-10 | 73 | 3.21E-11 | TEQGGAHFSVSSLAEGSVTSV  GSVNPAENFR |
| P13010 | 2449.264 | 2E-13 | 128 | 1.43E-14 | KYAPTEAQLNAVDALIDSMSLA  K |
| P13010 | 2465.259 | 0.000812 | 33 | 0.000412 | KYAPTEAQLNAVDALIDSMSLA  K |
| P13010 | 2499.211 | 1.83E-15 | 86 | 8.59E-17 | KKDQVTAQEIFQDNHEDGPTAK |
| P13010 | 1571.864 | 5.17E-05 | 40 | 2.05E-05 | GITEQQKEGLEIVK |
| P13010 | 1121.65 | 0.003724 | 36 | 0.002161 | KVITMFVQR |
| P13010 | 2243.021 | 5.14E-08 | 86 | 1.11E-08 | DQVTAQEIFQDNHEDGPTAK |
| P13010 | 1377.764 | 0.00186 | 32 | 0.001014 | ANPQVGVAFPHIK |
| P13010 | 1086.588 | 0.012835 | 42 | 0.03239 | LFQCLLHR |
| P13010 | 1073.631 | 0.02454 | 39 | 0.01695 | LTIGSNLSIR |
| P13010 | 1003.492 | 0.017817 | 52 | 0.01193 | QYMFSSLK |
| P13010 | 993.555 | 0.084188 | 29 | 0.06544 | VITMFVQR |
| P13010 | 1031.614 | 0.095886 | 34 | 0.07536 | TLFPLIEAK |
| P13010 | 1109.61 | 0.110971 | 27 | 0.08829 | LGGHGPSFPLK |
| P13010 | 977.5051 | 0.173506 | 43 | 0.1453 | EDIIQGFR |
| P12956 | 1073.566 | 0.022906 | 12 | 0.01566 | IMATPEQVGK |
| P12956 | 1388.69 | 7.34E-05 | 70 | 3E-05 | DIISIAEDEDLR |
| P12956 | 2509.25 | 8.18E-05 | 22 | 3.39E-05 | LGSLVDEFKELVYPPDYNPEGK |
| P12956 | 1807.955 | 5.1E-07 | 107 | 1.35E-07 | TFNTSTGGLLLPSDTKR |
| P12956 | 1418.735 | 0.000308 | 32 | 0.002012 | DTGIFLDLMHLK |
| P12956 | 1172.689 | 0.100497 | 29 | 0.07931 | QELLEALTK |
| P12956 | 1918.86 | 4.16E-06 | 42 | 1.32E-06 | IMLFTNEDNPHGNDSAK |
| P12956 | 1399.737 | 0.019094 | 37 | 0.01287 | KPGGFDISLFYR |
| P12956 | 1161.663 | 0.032095 | 40 | 0.02274 | ILELDQFK |
| P12956 | 1172.689 | 0.100497 | 29 | 0.07931 | KQELLEALTK |
| P12956 | 2034.117 | 2.01E-06 | 37 | 5.97E-07 | FDDPGLMLMGFKPLVLLK |
| P12956 | 1757.964 | 1.83E-06 | 83 | 5.39E-07 | QIILEKEETEELKR |
| P12956 | 2344.171 | 1.08E-05 | 31 | 3.71E-06 | NIPPYFVALVPQEEELDDQK |
| P12956 | 1703.814 | 8.89E-05 | 62 | 9.22E-05 | SDSFENPVLQQHFR |
| P12956 | 1172.689 | 0.100497 | 29 | 0.07931 | KQELLEALTK |
